# TRIM21 promotes astrocyte-mediated neuroinflammation in experimental autoimmune encephalomyelitis by stabilizing RGMa via K33-linked ubiquitination

**DOI:** 10.1186/s12974-026-03769-4

**Published:** 2026-03-19

**Authors:** Shaoru Zhang, Zijie Wang, Ruiqi Cheng, Xiaolin Yang, Anan Jiang, Li Zhu, Xinyue Qin

**Affiliations:** 1 Department of Neurology, The First Affiliated Hospital of Chongqing Medical University, No.1 Youyi Road, Yuzhong District, Chongqing, 400016 China; 2 Taizhou School of Clinical Medicine, The Affiliated Taizhou People’s Hospital of Nanjing Medical University, 366 Taihu Road, Taizhou, Jiangsu 225300 China; 3 Department of Rehabilitation Medicine, Key Laboratory of Physical Medicine and Precision Rehabilitation of Chongqing Municipal Health Commission, The First Affiliated Hospital of Chongqing Medical University, Chongqing, 400016 China; 4 Department of Ultrasound Medicine, The First Affiliated Hospital, Zhejiang University School of Medicine, Hangzhou, Zhejiang 31000 China; 5 Department of Neurology, Dazhou Central Hospital, Dazhou, Sichuan 635099 China

**Keywords:** RGMa, TRIM21, Ubiquitination, Astrocytes, Experimental autoimmune encephalomyelitis.

## Abstract

Abnormal astrocytes activation contributes to disease progression in multiple sclerosis (MS), yet the regulatory mechanisms are not fully understood. Here, we found that repulsive guidance molecule a (RGMa) was significantly upregulated in astrocytes in a mouse model of experimental autoimmune encephalomyelitis (EAE). RGMa promoted the release of key inflammatory factors such as C-C motif chemokine ligand 2 (CCL2) from astrocytes. Through protein interaction screening, we identified the E3 ubiquitin ligase, tripartite motif-containing (TRIM) protein 21 (TRIM21), as a interaction protein of RGMa. Mechanistically, TRIM21 enhanced the stability of RGMa by catalyzing K33-linked ubiquitination at lysine 238 of RGMa in vitro. Astrocyte-specific knockdown of TRIM21 decreased the expression of both RGMa and CCL2, thereby effectively alleviating neurological deficits, inflammatory infiltration, and demyelination in acute EAE. Over-expression of RGMa reversed this protective effect in primary astrocytes. Notably, Quisinostat disrupted this signaling cascade by downregulating TRIM21 and consequently attenuating RGMa-mediated CCL2 induction, at least in part through TRIM21-dependent mechanisms, thereby exhibiting therapeutic potential for acute EAE. These results suggest that TRIM21-mediated stabilization of RGMa via K33-linked ubiquitination may represent a pathway contributing to astrocyte-mediated neuroinflammation, and might offer a potential target for MS intervention.

**Supplementary Information:**

The online version contains supplementary material available at 10.1186/s12974-026-03769-4.

## Introduction

Multiple sclerosis (MS) is an autoimmune-mediated inflammatory demyelinating disease of the central nervous system (CNS) [1]. The two major clinical needs, promoting remyelination [2, 3] and blocking chronic inflammation [4], remain unmet. Therefore, a deeper understanding of MS mechanisms is needed.

Astrocytes, the most abundant glial population in the CNS, contribute significantly to MS pathogenesis [5, 6]. Recent research has revealed that astrocytes in MS lesions exhibit a biphasic phenotype. They exert neuroprotective functions in the acute phase; however, upon sustained exposure to microglia-derived factors such as tumor necrosis factor-α (TNF-α), they rapidly transform into reactive astrocytes, secreting large amounts of mediators including C-C motif chemokine ligand 2 (CCL2) [7, 8]. This process establishes self-reinforcing inflammatory amplification loop, thereby directly exacerbating demyelination and axonal injury [9, 10]. However, the extracellular cues and intracellular homeostatic regulators governing this phenotypic switch remain poorly defined.

Repulsive guidance molecule a (RGMa) is a glycosylphosphatidylinositol-anchored membrane protein [11] implicated in pro-inflammatory response in the experimental autoimmune encephalomyelitis (EAE) animal model of MS. Our previous work and other studies have shown that RGMa disrupts the blood-brain barrier and promotes immune cell infiltration [12, 13]. However, key aspects of RGMa biology, including the regulatory network governing its expression in astrocytes, its protein stability control, and its potential role in astrocytic inflammation, remain elusive. The ubiquitin-proteasome system (UPS) is a major pathway for controlling protein homeostasis, with E3 ubiquitin ligases determining substrate specificity [14]. In the context of neuroinflammation, dysregulation of specific E3 ligases, such as tripartite motif-containing (TRIM) protein 11 (TRIM11), RING finger 157 (RNF157), cellular inhibitors of apoptosis (cIAP1), has been shown to reshape the microenvironment [15–17]. However, it remains to be determined whether RGMa is regulated by a specific E3 ligase and whether such regulation is sufficient to promote reactive astrogliosis. Elucidating this pathway might reveal a druggable regulatory node for MS.

In this study, by integrating clinical samples, primary astrocyte cultures, and EAE mouse models, we investigated mechanisms governing RGMa in astrocytes during the acute phase of EAE. We found that the E3 ligase TRIM21 stabilized RGMa in primary astrocytes. Notably, while TRIM21 has been shown to regulate astrocyte metabolism via pyruvate kinase M2 (PKM2) in EAE [18], our findings reveal a distinct mechanism whereby TRIM21 stabilizes RGMa through K33-linked polyubiquitination. These observations suggest that TRIM21 may orchestrate diverse astrocyte responses through multiple substrates. Mechanistically, TRIM21-mediated RGMa stabilization facilitates astrocyte activation and CCL2 secretion, contributing to neuroinflammation and demyelination. Furthermore, we found that Quisinostat, a histone deacetylase inhibitor (HDACi) with blood-brain barrier**-**penetrating properties [19, 20], inhibited TRIM21. Administration of Quisinostat effectively attenuated EAE progression. These results indicate that TRIM21-mediated RGMa stabilization contributes to astrocyte activation during acute-phase EAE, warranting further investigation as a potential therapeutic target.

## Materials and methods

### Human specimens

We enrolled MS patients as the disease group and age- and sex-matched volunteers as controls. Ethics approval was obtained from the First Affiliated Hospital of Chongqing Medical University Ethics Committee (approval No. 2023-046). Written informed consent was obtained from participants before any procedure. Peripheral venous blood was drawn prior to the first intravenous corticosteroid administration, either at outpatient visits or on hospital admission. Serum RGMa levels were quantified with a commercial enzyme-linked immunosorbent assay (ELISA) kit (Human RGMa DuoSet ELISA, DY2459-05, R&D Systems, Minnesota, USA) following the manufacturer’s instructions. Peripheral-blood mononuclear cells (PBMCs) were isolated by density-gradient centrifugation on Ficoll-Paque PLUS (P9011, Solarbio, Beijing, China).

### Bioinformatics analysis

The microarray dataset GSE138614 was retrieved from the Gene Expression Omnibus (GEO) repository of the National Center for Biotechnology Information (https://www.ncbi.nlm.nih.gov/geo/). Raw CEL files from control and MS samples were imported into R version 4.2.3, background-corrected, normalized, and summarized using the robust multi-array average algorithm implemented in the limma package. After probe-to-gene annotation, the expression of TRIM21, which showed significant interaction with RGMa in our mass-spectrometry screen, was compared between groups.

### Animals

Mice were raised and maintained at the animal research center of Chongqing Medical University. All animal procedures in the project were approved by Institutional Animal Care and Use of Chongqing Medical University (IACUC-CQMU-2023-0392). Experimental procedures strictly followed institutional guidelines. Animals were housed under a 12-hour light-dark cycle at controlled temperature and humidity, with ad libitum access to food and water. The mice were randomly divided into groups.

### Myelin oligodendrocyte glycoprotein peptide 35–55 (MOG_35−55_)-induced EAE model

The details of EAE model construction were as previously described [21]. Briefly, 200 µL of PBS solution containing 0.35 mg MOG_35−55_ peptide (HY-P1240, MedChemExpress, NJ, USA) was added to 200 µL Freund’s complete adjuvant containing 10 mg/ml *mycobacterium tuberculosis* (Mtb, BD, 231141, NJ, USA) and the mixture was efficiently emulsified. Each mouse was immunized subcutaneously adjacent to the spine with 200 µL of the emulsion (total of 175 µg MOG_35−55_ and 1 mg Mtb per mouse) and subsequently received an intraperitoneal injection of 200 ng pertussis toxin (PTX, 181, List Biological Labs, CA, USA) on days 0 and 2 post-immunization (total of 400 ng PTX per mouse).

### Evaluation of the clinical course and tissue processing

The clinical assessment of EAE mice involved daily recording of body weight and paralysis severity, scored according to the following scale: 0 (no symptoms); 1 (flaccid tail paralysis); 2 (hindlimb paresis); 3 (hindlimb paralysis); 4 (tetraplegia); 5 (moribund or death due to EAE) [22]. Where appropriate, mice were assigned intermediate scores (0.5, 1.5, 2.5, 3.5) to capture subtler changes in clinical severity. All mice were humanely euthanatized 28 days post-immunization (dpi). For molecular analysis, mice were transcardially perfused with phosphate-buffered saline (PBS) under deep anesthesia; the brain and spinal cord were rapidly isolated and quickly frozen in liquid nitrogen, and stored at-80 °C. For histological analysis, separate cohorts were perfused with 4% paraformaldehyde (PFA); tissues were embedded in paraffin, and 5-micrometer (µm) sections were prepared for histochemical assessment.

### Stereotaxic injection

To selectively knock down TRIM21 in astrocytes under the gfaABC1D promoter, Adeno-associated virus (AAV) 9 vectors expressing shTRIM21 or control shRNA were delivered via intracerebroventricular (icv.) injection into the lateral ventricle. AAV9-gfaABC1D-shTRIM21-EGFP (AAV-shTRIM21), negative control shRNA (AAV-shNC), AAV9-gfaABC1D-RGMa-EGFP (AAV-RGMa) and AAV9 vector for RGMa (AAV-vector) were constructed by Hanheng Biotechnology (Hanheng Biotechnology Co., Ltd., Shanghai, China). Anesthetized mice were secured to a brain stereotaxic apparatus (RWD Life Science, Shenzhen, China). A mid-line scalp incision was made along the anterior border of the head. Coordinates relative to the skull surface at Bregma were as follows: anteroposterior (AP) -0.22 mm, mediolateral (ML) ± 1.0 mm, and dorsoventral (DV) -2.8 mm [23]. A single icv. injection of 2 µL AAV was administered, followed by a 5-minute microsyringe hold before slowly withdrawal. The wound was sutured, and penicillin powder was applied topically. EAE model was established 28 days after icv. injection.

### Administration of quisinostat *in vivo*

After 10 days of induction with MOG₃₅-₅₅ by which time mice gradually exhibited tail weakness, the mice were randomly assigned to receive daily intraperitoneal injections of either Quisinostat (HY-12726, MedChemExpress, USA) at 10 mg/kg or an equal volume of vehicle control for 2 weeks.

### Histological analysis

Hematoxylin-eosin (HE) staining was used to evaluate inflammatory cellular infiltration. After ethanol dewaxing and hydration, the 5-µm sections underwent HE staining followed by dehydration and mounting. Luxol Fast Blue (LFB) staining was used to evaluate demyelination severity. After dewaxing and hydration, sections were incubated with LFB solution, followed by counterstaining with alcoholic eosin solution. The inflammatory scores of HE were calculated as follows: 0 (no inflammation); 1 (perivascular and meningeal infiltration); 2 (infiltration involving less than 1/3 white matter); 3 (infiltration involving more than 1/3 white matter); 4 (extensive white matter infiltration) [24]. The demyelinating scores of LFB were calculated as follows: 0 (normal); 1 (scattered myelin damage); 2 (focal demyelination); 3 (confluent perivascular or subcortical demyelination); 4 (large-area demyelination involving half of the spinal cord); 5 (extensive demyelination involving the entire spinal cord) [25]. Images were captured using a Zeiss conventional microscope, and ImageJ software was employed for further analysis.

### Cell culture and reagents

Cerebral cortices from 1 to 3 day-old neonatal mice were rapidly dissected, carefully stripped of meninges, and washed three times with ice-cold Dulbecco’s Modified Eagle Medium (DMEM)/F12 medium (PM150310, Procell, Wuhan, China) containing 1% penicillin/streptomycin (P/S; 15140-122, Gibco, NY, USA). Then the tissues were digested in 0.25% trypsin-EDTA and DNase I (1 mg/ml) for 15 min. Trypsinization was terminated by adding DMEM/F12 medium supplemented with 10% fetal bovine serum (FBS, F0193, Sigma-Aldrich, MO, USA), 1% P/S and with 5 ng/ml Heparin-binding epidermal growth factor-like growth factor (HBEGF, HY-P7194, MedChemExpress, NJ, USA). Tissues were triturated and filtered through a 70-µm cell strainer to obtain a single-cell suspension, then seeded onto poly-L-lysine-coated dishes. After centrifugation at 500 × g for 10 min at 4 °C, cells were resuspended in DMEM/F12 containing 10% FBS, 1% P/S, and 5 ng/ml HBEGF, and cultured at 37 °C in incubator with 5% CO_2_, with medium replaced every 3 days. Detached microglia were removed by tapping the dishes and discarding the supernatant. To eliminate residual microglia, sequential shaking was performed (30 min at 180 rpm followed by > 2 h at 220 rpm). Astrocyte purity (> 95%) was confirmed by immunostaining for the astrocytic marker glial fibrillary acidic protein (GFAP; Fig. S1). After reaching full coverage (days 7–10), astrocytes were trypsinized with 0.06% trypsin-EDTA seeded in 6-well plates and cultured for further treatments [26–28].

HEK293T cells and SVG p12 cells were purchased from the Cell Bank of the Chinese Academy of Sciences (Shanghai, China) and cultured in DMEM containing 10% FBS and 1% P/S at 37 °C in the incubator with 5% CO_2_. Where indicated, astrocytes were treated with 50 ng/ml TNF-α (HY-P7058, MedChemExpress, NJ, USA) and Interleukin-1β (IL-1β, HY-P7073, MedChemExpress) or vehicle for 24 h. Cells were treated with 50 µg/ml Cycloheximide (CHX, HY-12320, MedChemExpress, NJ, USA) for the time indicated, or treated with 20 µM MG132 (HY-13259, MedChemExpress, NJ, USA), Quisinostat (HY-12726, MedChemExpress, NJ, USA). The optimal concentration of Quisinostat was determined by Cell Counting Kit-8 (CCK-8) assay (MA0218-5, MeilunBio, Dalian, China) according to the manufacturer’s instruction.

### Gene cloning and plasmid construction

The complementary DNA (cDNA) encoding RGMa, TRIM21 and ubiquitin (Ub) was amplified from HEK293T cell cDNA by standard PCR. PCR products were validated by agarose gel electrophoresis and purified using a PCR purification kit (EG101-01, Transgen, Beijing, China). Purified fragments were cloned into epitope-tagged expression vectors via homologous recombination using the pEASY^®^-Basic Seamless Cloning and Assembly Kit (CU201-02, Transgen, Beijing, China). After Sanger sequencing validation (Tsingke Biotechy, Beijing, China), the recombinant plasmids were transformed into E. coli DH5α cells (CD201-01, Transgen, Beijing, China). Plasmids were then extracted using TlANprep Midi Plasmid Kit (DP106-02, TIANGEN, Beijing, China) after overnight culture with shaking. On the basis of successfully constructed plasmids, mutants of RGMa, TRIM21, and ubiquitin were generated using specific primers. The primer sequences are detailed in the supplementary Table 1.

### *In vitro* lentivirus (LV) transduction and plasmids transfection

LV-Vector, LV-TRIM21, LV-negative control shRNA (LV-shNC), and LV-shRNA-RGMa (LV-shRGMa) were packaged by Hanheng Biotechnology (Hanheng Biotechnology, Shanghai, China). Plasmid transfection was performed using Lipofectamine 3000 reagent (L3000015, Invitrogen, CA, USA) according to the manufacturer’s guidelines.

### Co-immunoprecipitation (Co-IP) assays

For Co-IP assays, cell lysates were mixed with the normal IgG or indicated primary antibody protein and A/G magnetic beads (HY-K0202, MedChemExpress, NJ, USA). The immunocomplexes were rotated slowly and incubated overnight at 4 °C. After washing five times with lysis buffer, the immunocomplexes were finally analyzed by western blotting assays, using the indicated primary antibodies and appropriate HRP-conjugated secondary antibodies. The antibodies used for Co-IP analysis are listed in Supplementary Table 2.

### Immunoprecipitation-mass spectrometry (IP-MS) analysis

The cell lysates were centrifuged at 12,000 × g to obtain supernatants, which were incubated with anti-RGMa agarose beads for 2 h at 4 °C. After washing, immunoprecipitated proteins were separated by SDS-PAGE and subjected to in-gel digestion followed by mass spectrometry analysis to identify RGMa-interacting proteins.

### Ubiquitylation assays and prediction of ubiquitination sites

For in vitro RGMa ubiquitination assays, Myc-RGMa, Flag-TRIM21, and HA-ubiquitin were transfected into HEK293T cells using Lipofectamine 3000. After 24 h, cells were harvested and lysed in lysis buffer. Cell extracts were immunoprecipitated with an anti-Myc antibody, washed five times with the lysis buffer, and analyzed using immunoblotting with anti-HA monoclonal antibody. GPS-Uber was utilized to predict putative ubiquitination sites.

### Ribonucleic acid (RNA) isolation and reverse-transcription quantitative real-time PCR (RT-qPCR)

Total RNA was extracted from PBMCs, cells and mouse brain/spinal cord tissues using RNAiso Plus reagent (9109, Takara, Otsu, Shiga, Japan) according to the manufacturer’s protocol. After RNA purification and quantification, cDNA was synthesized using a reverse transcription kit (R333, Vazyme, Nanjing, China). Quantitative PCR was performed with SYBR Green Master Mix kit (Q711,Vazyme, Nanjing, China), and messenger RNA (mRNA) relative expression levels were normalized against β-actin or glyceraldehyde-3-phosphate dehydrogenase (GAPDH) and quantified using the 2^−ΔΔCT^ method. The primer sequences are detailed in the Supplementary Table 3.

### Western blotting

For immunoblotting assays, cells or mouse tissues were homogenized in RIPA lysis buffer. Equal amounts of protein were separated by SDS-PAGE and transferred to PVDF membranes (ISEQ00010-N2, Merck Millipore, Darmstadt, Germany). Membranes were blocked with 5% skim milk and incubated at 4 °C overnight with primary antibodies. After three washes with TBST, membranes were incubated with HRP-labeled secondary antibodies at room temperature for 1 h, and protein bands were developed using ECL reagent (BMU102, Abbkine, Wuhan, China). ImageJ software was used for image analysis and quantification of band density. Antibodies used are listed in Supplementary Table 4.

### Immunofluorescence and colocalization analysis

For tissue immunofluorescence analysis, the sections were processed by dewaxing, hydration, peroxidase blocking, and antigen retrieval. Subsequently, the sections were incubated overnight at 4 °C with primary antibodies, followed by secondary antibody incubation at room temperature. Images were acquired using the LAX S imaging system (Leica, Germany). For cellular immunofluorescence and colocalization assays, cells grown on confocal dishes were transfected with plasmids encoding Flag-TRIM21 and Myc-RGMa. The cells were then fixed with 4% PFA and permeabilized with 0.3% Triton X-100. After blocking, cell samples were incubated with relevant primary antibodies overnight at 4 °C, followed by secondary antibody incubation. Images were acquired using a confocal microscope (Nikon, Japan). Antibodies used for immunofluorescence analysis were detailed in Supplementary Table 5.

### Statistical analysis

Statistical analyses were carried out employing GraphPad Prism software (v10.1; GraphPad Software, CA, USA) and R software (version 4.2.3). The schematic diagram was created by Figdraw (www.figdraw.com). Data normality was assessed using the Shapiro-Wilk test. Normally distributed data were expressed as mean ± SD. Data were from at least three independent experiments using biological replicates (individual animals or independent cell cultures).

For two-group comparisons, if the data were normally distributed and exhibited homogeneity of variance (assessed by Levene’s test), an independent samples t-test was used; if the data were normally distributed but variances were unequal, Welch’s t-test was employed; if the data did not follow a normal distribution, the Mann-Whitney U test was applied.

For four-group comparisons, one-way ANOVA was performed when normality and equal variance assumptions were met, followed by Tukey’s HSD test. When normality was retained but variances differed, Welch’s ANOVA was adopted, with Games-Howell post hoc. For non-normal data, the data were analyzed by the Kruskal-Wallis H test followed by Dunn’s test.

For two-way ANOVA, when normality and homogeneity of variance assumptions were met, Tukey’s post hoc test was applied. If the data were non-normally distributed or variances were heterogeneous, an Aligned Rank Transform ANOVA was applied, followed by contrast tests on the ranked data.

All statistical analyses were two-tailed, with statistical significance defined as *p* < 0.05. Significance levels in figures were indicated as *p < 0.05, **p < 0.01, ***p < 0.001, and ****p < 0.0001; ns, not significant.

## Results

### RGMa exacerbates neuroinflammation by potentiating the pro-inflammatory response of astrocytes in MS/EAE

**Fig. 1:**
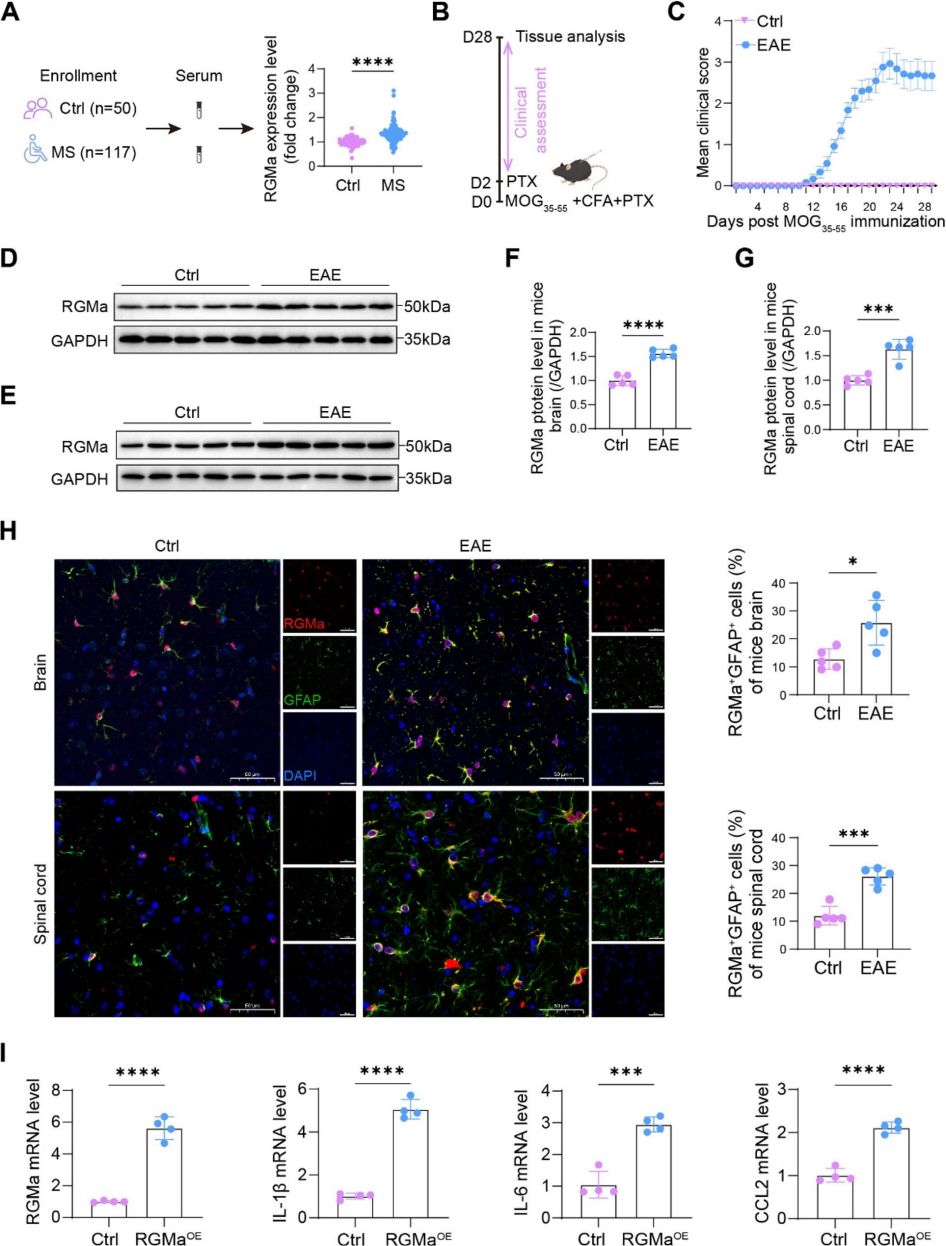
RGMa is upregulated in MS patients and EAE mice and localizes to astrocytes. **A** RGMa levels in peripheral blood samples from MS patients (n = 117) and controls (n = 50), as quantified by ELISA. **B** Schematic depicting the time course of MOG35-55-induced EAE in the murine model. **C** Clinical scores of EAE mice following immunization with MOG35-55 peptide (n = 12). **D** Representative Western blot analysis of RGMa protein expression in brain lysates from EAE mice at 28 dpi (n = 5). **E** Corresponding Western blot analysis of RGMa expression in spinal cord lysates from EAE mice at 28 dpi (n = 5). **F** Quantitative assessment of RGMa protein expression in brain lysates, normalized to GAPDH (n = 5). **G** Quantification of RGMa expression in spinal cord lysates, normalized to GAPDH (n = 5). **H** Immunofluorescence analysis illustrating the spatial distribution and co-localization of RGMa (red) with the astrocyte marker glial fibrillary acidic protein (GFAP, green) in brain and lumbar spinal cord sections between control and EAE mice at 28 dpi (n = 5). Note: IgG isotype controls confirmed staining specificity (Fig. S2). Scale bar, 50 µm. DAPI, 4',6-Diamidino-2'-phenylindole. **I** Inflammatory cytokine mRNA expression levels of IL-1β, IL-6, and CCL2 in primary astrocytes at 24 hours after lentivirus-mediated overexpression of RGMa or vector control

To explore the functional significance and clinical relevance of RGMa in MS, we first quantified serum RGMa levels by ELISA in a cohort of MS patients and controls. RGMa concentrations were significantly higher in MS patients than in controls (Fig. 1A), corroborating our previous observations [13]. Subsequently, we employed the MOG_35−55_-induced EAE model (Fig. 1B). Mice developed varying degrees of neurological impairment from 10 dpi (Fig. 1C), accompanied by a marked upregulation of RGMa protein in both brain and spinal cord tissues (Fig. 1D-G). Immunofluorescence staining further revealed a substantial enhancement of RGMa signal within astrocytes in the brain and spinal cord of EAE mice, suggesting elevated RGMa expression in astrocytes during EAE pathogenesis (Fig. 1H). IgG isotype controls confirmed staining specificity (Fig. S2).

Reactive astrogliosis is a hallmark pathological event in MS lesions, and the pro-inflammatory mediators released by activated astrocytes are recognized as a key mechanism driving lesion expansion [7]. To evaluate whether RGMa contributes to this process, primary cortical astrocytes were transduced with overexpression or knockdown of RGMa and subsequently challenged with TNF-α plus IL-1β for 24 h. (For purity assessment of astrocyte cultures, see Fig. S1). Upon cytokine stimulation, astrocytes exhibited an upregulation of key pro-inflammatory mediators, including IL-1β, IL-6 and CCL2, as measured by qRT-PCR (Fig. 1I and Fig. S3). The inflammatory phenotype observed in our primary cultures was attributed to astrocytes. First, our cultures were highly enriched for the GFAP marker (Fig. S1), making it unlikely that contaminating cells drove the consistent, high-magnitude changes in gene expression. Second, the coordinated regulation of IL-1β, IL-6 and CCL2 across all gain- and loss-of-function manipulations of RGMa indicated a common regulatory mechanism within a homogeneous cell population. Collectively, these data suggest that RGMa may exacerbate neuroinflammation by potentiating astrocytic activation.

### The E3 ligase TRIM21 interacts with RGMa

**Fig. 2:**
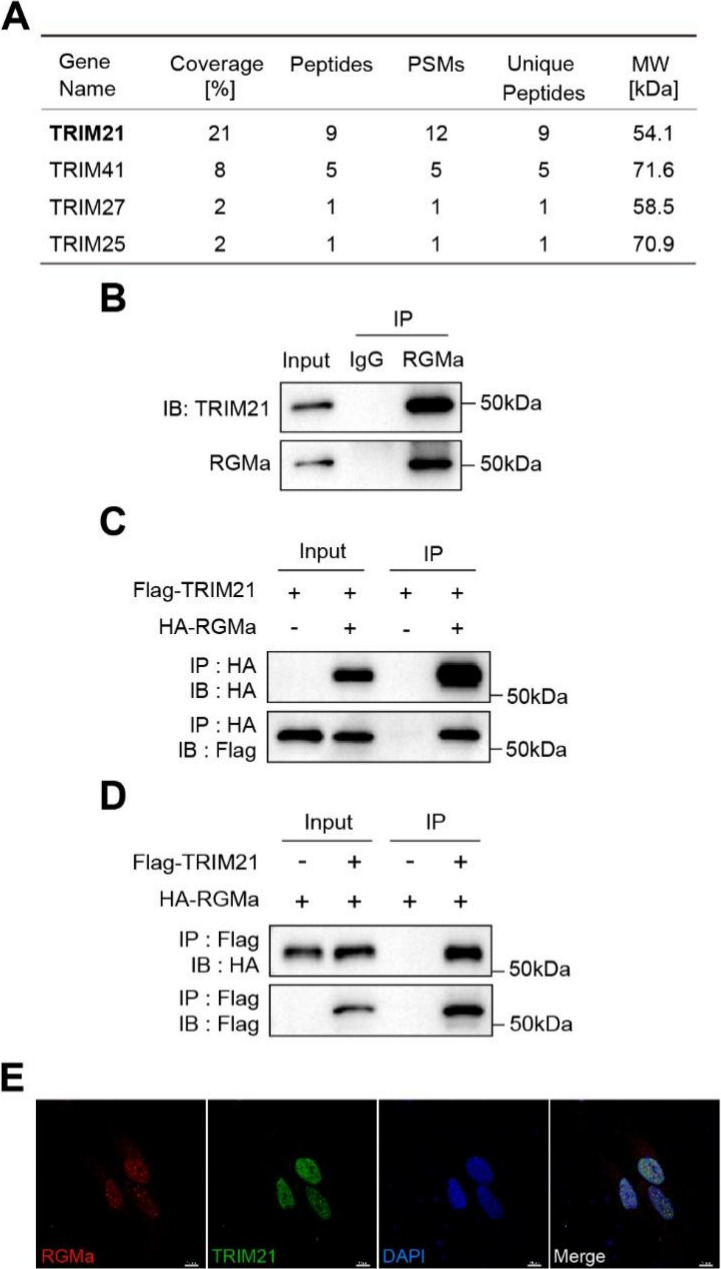
TRIM21 interacts with RGMa and exhibits overlapping distribution in astrocytes. **A** IP-MS screening identified TRIM21 as a candidate RGMa-interacting protein in astrocytes. **B** Endogenous co-immunoprecipitation of TRIM21 and RGMa from astrocyte lysates. **C**, **D** Reciprocal Co-IP of exogenous Flag-TRIM21 and HA-RGMa transiently co-expressed in HEK293T cells confirmed the interaction between TRIM21 and RGMa. **E **Representative confocal micrograph showing co-localization of RGMa (red) and TRIM21 (green) within GFAP+ astrocytes. Scale bar, 10 µm

To explore proteins that interact with RGMa, we performed IP-MS to identify proteins associated with RGMa. IP-MS analysis indicated the presence of TRIM family members (a class of E3 ubiquitin ligases), including TRIM21 and TRIM41, in the RGMa immunoprecipitation complex; of these, TRIM21 exhibited the highest-confidence interaction with RGMa (Fig. 2A), prompting us to focus on TRIM21. Given the established role of ubiquitination in governing protein stability, localization, and interactions, we sought to determine whether TRIM21 interacted with RGMa. Endogenous Co-IP from primary astrocyte lysates confirmed that TRIM21 co-precipitates with RGMa (Fig. 2B). To rule out antibody cross-reactivity, we next over-expressed epitope-tagged plasmids. HEK293T cells were co-transfected with Flag-TRIM21 and HA-RGMa. Anti-Flag beads immunoprecipitated HA-RGMa only when Flag-TRIM21 was present (Fig. 2C and D), confirming an exogenous interaction between TRIM21 and RGMa. Finally, we examined subcellular distribution. Primary astrocytes co-transfected with Myc-RGMa and Flag-TRIM21 were stained and imaged by confocal microscopy. Both proteins exhibited punctate signals that overlapped within astrocytes (Fig. 2E).

### TRIM21 post-transcriptionally stabilizes RGMa via the ubiquitination

**Fig. 3:**
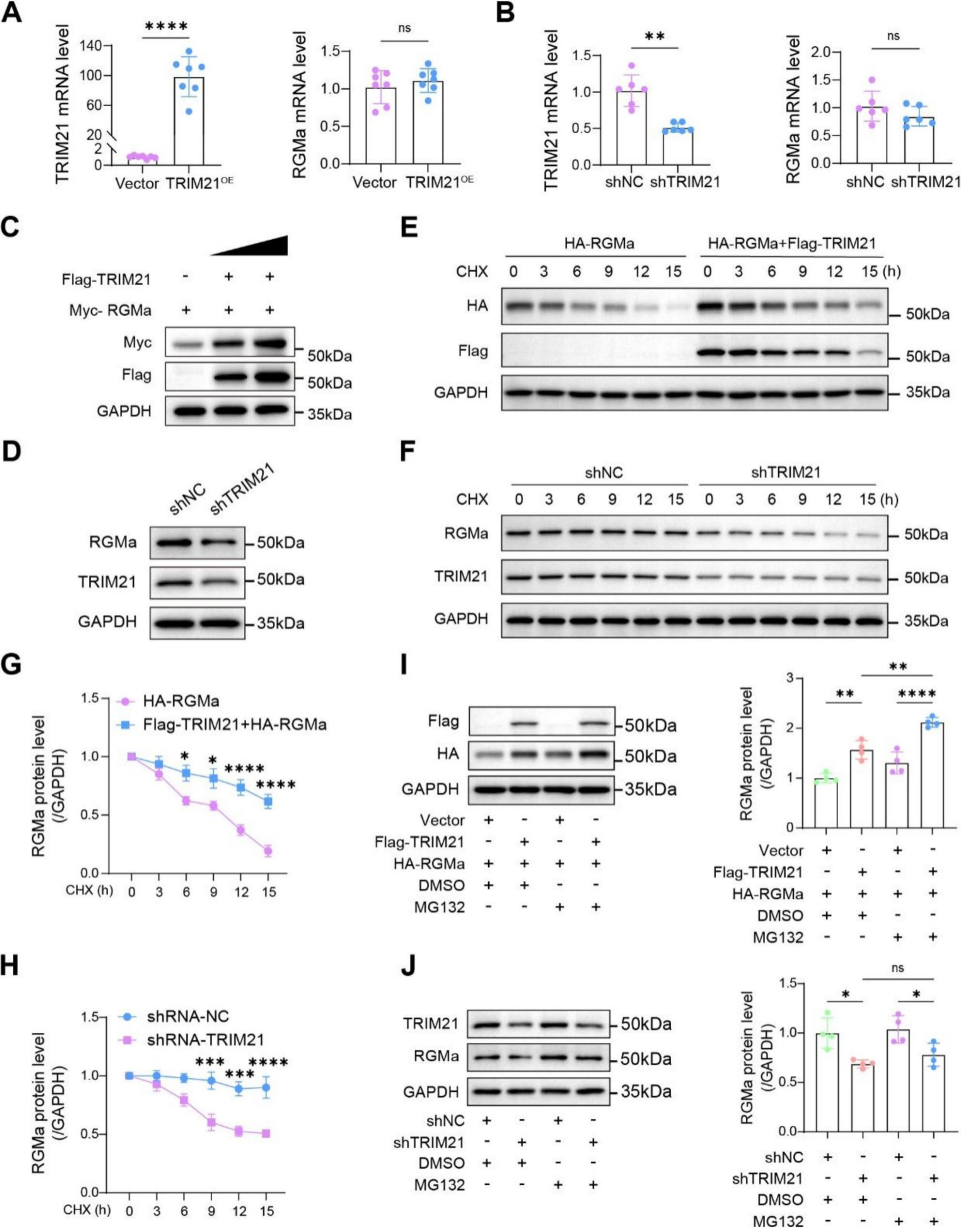
The E3 ubiquitin ligase TRIM21 mediates RGMa protein stabilization. **A** mRNA expression of TRIM21 and RGMa in astrocytes following TRIM21 overexpression. **B** mRNA levels of TRIM21 and RGMa in astrocytes transfected with negative control shRNA (shNC) plasmids or TRIM21-targeting (shTRIM21) shRNA plasmids. **C** Immunoblot analysis of Myc-RGMa co-expressed with increasing amounts of Flag-TRIM21 in HEK293T cells. **D** RGMa protein expression in astrocytes transduced with shNC or shTRIM21. **E** Cycloheximide (CHX) chase assay in HEK293T cells expressing HA-RGMa alone or with Flag-TRIM21 to assess RGMa protein stability. **F** CHX chase assay in HEK293T cells treated with shNC or shTRIM21. **G**-**H** Quantification of RGMa protein levels from experiments in **E** and **F**. **I ** Western blot of astrocytes co-expressing Flag-TRIM21 and HA-RGMa and treated with DMSO or the proteasome inhibitor MG132. **J** RGMa protein levels in astrocytes transfected with shNC or shTRIM21 and treated with DMSO or MG132

To rule out the possibility that TRIM21 regulates RGMa at the transcriptional level, we performed RT-qPCR in primary astrocytes. Neither TRIM21 knockdown (12, 24, 48, 72 h) nor overexpression (12, 24, 48, 72 h) altered RGMa mRNA at any time point (Fig. 3A and B and Fig. S4), indicating post-transcriptional regulation. We next determined steady-state of RGMa protein levels. Exogenous TRIM21 elevated RGMa in a dose-dependent manner (Fig. 3C). Conversely, short hairpin RNA (shRNA) targeting TRIM21 reduced RGMa protein levels in astrocytes (Fig. 3D). Subsequently, to test whether TRIM21 controls RGMa stability, we blocked new protein synthesis with 50 µg/ml CHX and monitored RGMa decay by western blot. Compared with the control group, overexpression of TRIM21 significantly prolonged the half-life of RGMa protein (Fig. 3E and G), whereas knockdown of TRIM21 by shRNA accelerated RGMa degradation (Fig. 3F and H). Moreover, MG132 treatment further increased RGMa when TRIM21 was already elevated and partially rescued the reduction caused by TRIM21 silencing (Fig. 3I and J). Collectively, these data suggest that TRIM21 stabilizes the RGMa protein in astrocytes and HEK293T cells, most likely through its E3 ubiquitin-ligase activity, as evidenced by: (i) the significant accumulation of RGMa upon MG132 treatment in TRIM21-overexpressing cells, and (ii) the trend (though not statistically significant) toward recovery of RGMa levels with MG132 in TRIM21-silenced cells, which is consistent with the antagonism of proteasomal degradation but also suggests that RGMa may be partially degraded through alternative pathways when TRIM21 was knockdown (e.g., autophagy), thereby reducing the salvage effect of MG132.

### TRIM21 stabilizes RGMa through K33-linked polyubiquitination

**Fig. 4:**
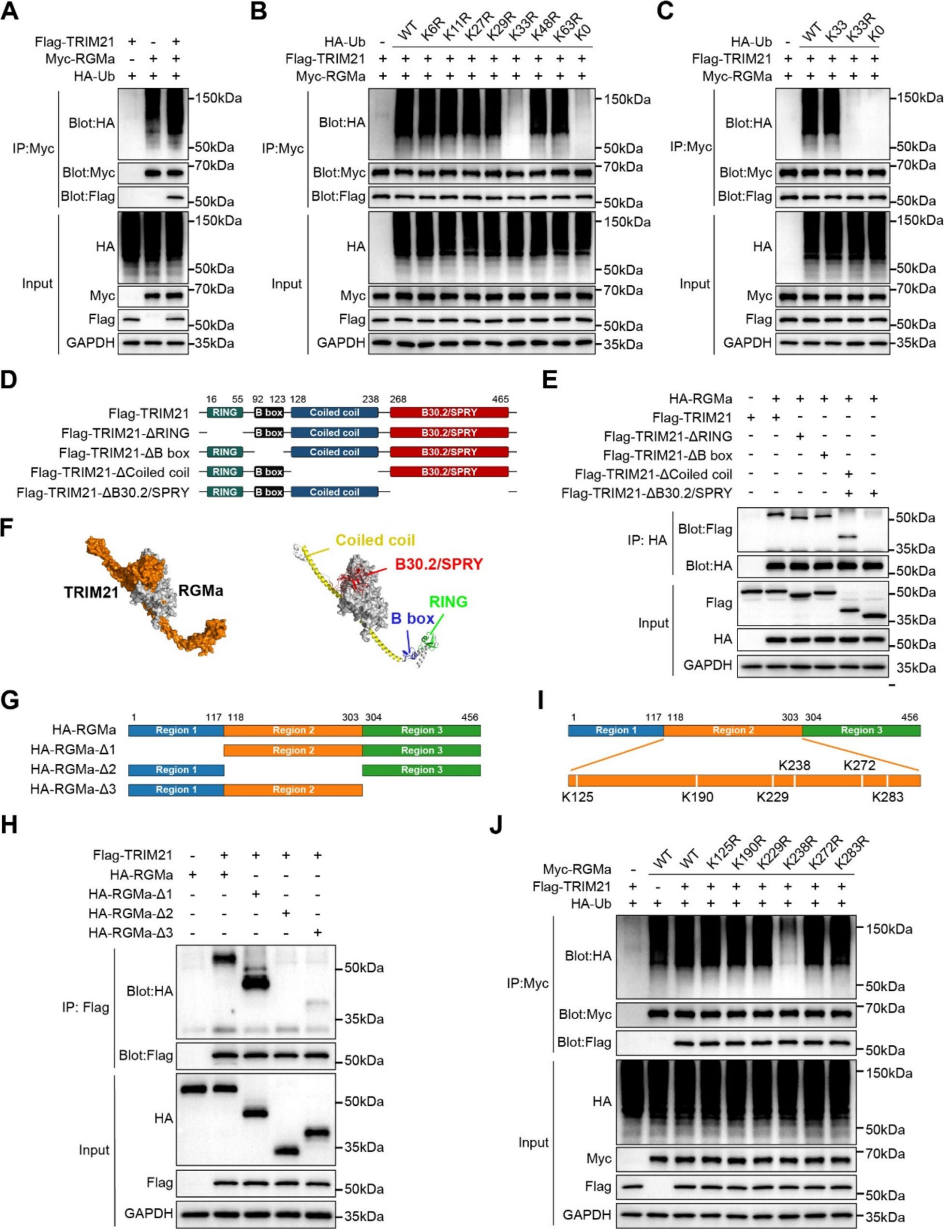
TRIM21 catalyzes K33-linked polyubiquitination of RGMa. **A** Ubiquitination assay in HEK293T cells co-expressing Myc-RGMa, Flag-TRIM21, and HA-Ub. Immunoprecipitation of Myc-RGMa followed by immunoblotting.**B** Co-immunoprecipitation (Co-IP) analysis of HEK293T cells expressing Flag-TRIM21 and Myc-RGMa in the presence of either wild-type (WT) HA-Ub or its series of HA-tagged lysine-to-arginine (KR) ubiquitin mutants.**C** Analysis of RGMa ubiquitination in HEK293T cells co-expressing Flag-TRIM21 and Myc-RGMa together with WT ubiquitin, a ubiquitin mutant retaining only K33, a K33-to-arginine (K33R) mutant, or a lysine-null (K0) ubiquitin. **D** Schematic depicting the domain architecture of TRIM21 and its truncation mutants. **E** Assessment of RGMa ubiquitination by full-length TRIM21 and its truncation mutants. **F** Structural prediction by AlphaFold molecular docking revealed an interaction interface between RGMa and TRIM21. The model predicted multiple TRIM21 domains, including the coiled-coil (yellow), B-box (blue), RING (green), and B30.2/SPRY (red) regions, defining the binding interface as shown in the PyMOL visualization. **G** Schematic illustrating the domain structure of RGMa and its truncation variants. **H** Ubiquitination assay in HEK293T cells co-expressing Flag-TRIM21 with either full-length HA-RGMa or its truncation mutants. **I** Schematic mapping the identified ubiquitination sites on RGMa. **J** Ubiquitination analysis of Myc-RGMa or its ubiquitination-site mutants co-expressed with Flag-TRIM21 and HA-Ub in HEK293T cells. Myc immunoprecipitates were immunoblotted with the indicated antibodies

As TRIM21 is an E3 ubiquitin ligase, we investigated whether it regulates the ubiquitination of RGMa. Co-transfection of Flag-TRIM21 with HA-ubiquitin (HA-Ub) and Myc-RGMa into HEK293T cells showed that TRIM21 overexpression promoted RGMa ubiquitination (Fig. 4A). To determine the ubiquitin chain catalyzed by TRIM21 on RGMa, each lysine (K) residue in HA-Ub (K6, K11, K27, K29, K33, K48, and K63) was mutated to arginine (R), and all lysine residues were mutated to arginine (K0). The wild type (WT) HA-Ub and its mutants were co-transfected with Flag-TRIM21 and Myc-RGMa. Strikingly, only K33R blocked TRIM21-mediated RGMa poly-ubiquitination (Fig. 4B), similarly to the ubiquitin mutant K0 (Fig. 4C). The mutant retaining only lysine 33 (K33) was capable of forming ubiquitin chains on RGMa that closely resembled wild-type ubiquitin (Fig. 4C). These results suggested that TRIM21 stabilizes RGMa by promoting its K33-linked polyubiquitination.

TRIM21 comprises a Really Interesting New Gene (RING) domain (residues 16–55), a B-box domain (residues 92–123), a coiled-coil domain (residues 128–238), and a B30.2/SPRY domain (residues 268–465) (Fig. 4D). To map the domain required for its interaction with RGMa, a series of Flag-TRIM21 truncation mutants were generated based on its distinct structural regions. Co-IP assays following co-transfection with HA-RGMa revealed that the B30.2/SPRY domain of TRIM21 was necessary for binding to RGMa (Fig. 4E), a finding supported by molecular docking analysis of AlphaFold2 [29] that confirmed the conformational interaction between the two proteins (Fig. 4F). We next constructed three truncation mutants of RGMa (Fig. 4G) and assessed their ability to bind TRIM21. TRIM21 interacted strongly with RGMa-Δ1 (Δ1–17) and RGMa-Δ3 (Δ304–456), but not with RGMa-Δ2 (Δ118–303), indicating that residues 118–303 of RGMa were required for TRIM21 binding (Fig. 4H). Together, these findings provide mechanistic insight into the specific molecular interaction between TRIM21 and RGMa.

To further identify the specific lysine residue(s) in RGMa that are ubiquitinated by TRIM21, six candidate lysines, predicted by the online tool GPS-Uber (https://gpsuber.biocuckoo.cn/index.php. Accessed on 26 Jun 2025) were mutated to arginine. Each of these six lysine (K) residues was individually mutated to arginine (R) (Fig. 4I). Myc-RGMa or its lysine mutants were co-expressed in HEK293T cells together with HA-Ub and Flag-TRIM21. Compared with WT Myc-RGMa and other mutants, the K238R mutant exhibited markedly reduced ubiquitination (Fig. 4J), suggesting that K238 was a potential ubiquitination site on RGMa targeted by TRIM21. CHX chase assays further showed that K238R accelerated RGMa degradation compared to wild-type in the presence of TRIM21 (Fig. S5), indicating that K238 is critical for TRIM21-mediated stabilization. These results suggest that TRIM21 stabilizes RGMa by promoting K33-linked polyubiquitination at K238, as determined in cellular models.

### TRIM21 expression is elevated in MS and EAE

**Fig. 5:**
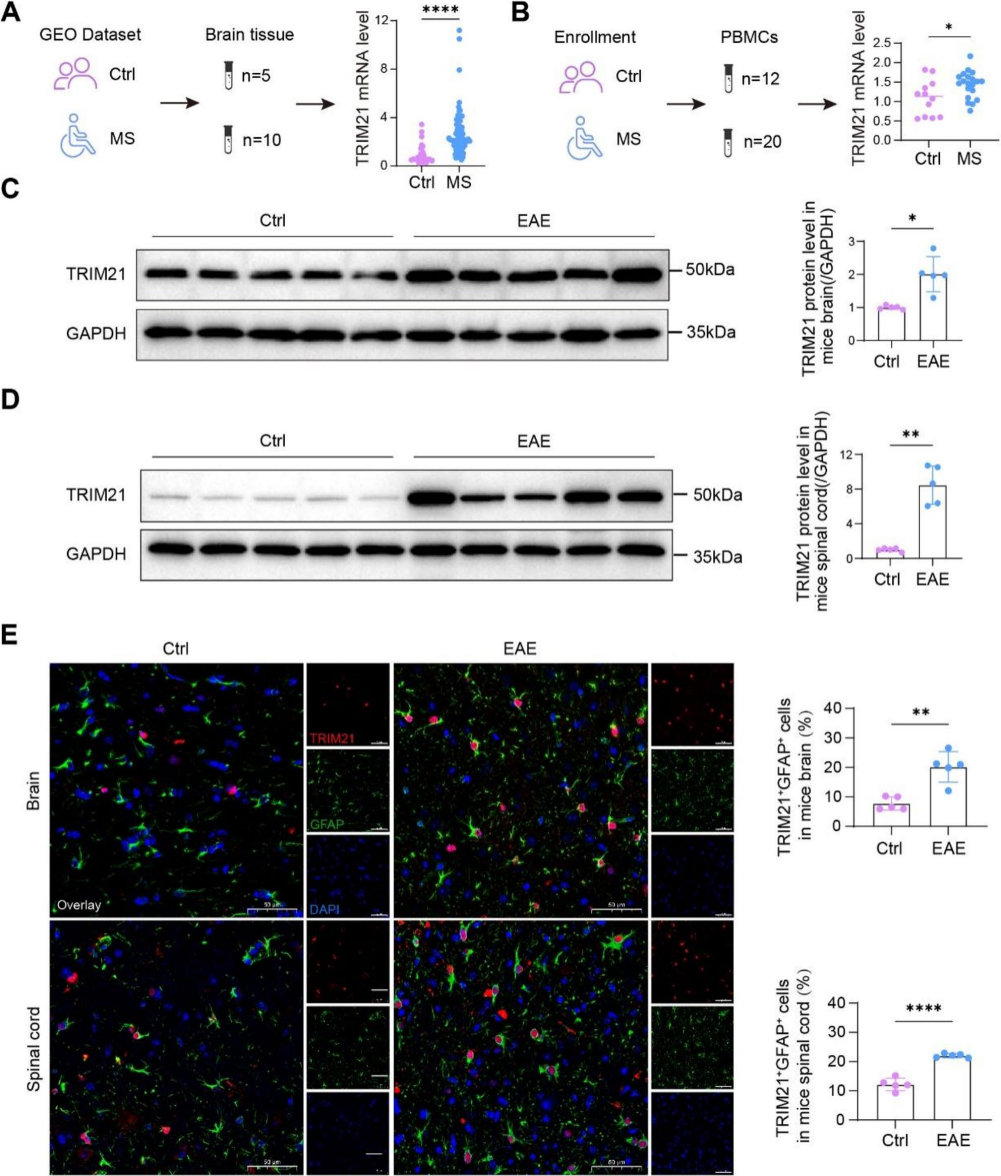
TRIM21 is elevated in astrocytes across MS. **A** Analysis of TRIM21 mRNA expression in the control and white matter lesion tissues of MS patients based on gene expression profiling microarray data (GSE138614). **B** TRIM21 mRNA levels in PBMC samples of healthy controls (n = 12) and MS patients (n = 20) were quantified by RT-qPCR. **C** Representative immunoblots and corresponding quantification of TRIM21 protein levels in brain tissues from EAE mice at 28 dpi (n = 5). **D** Representative immunoblot images and the corresponding quantitative analysis of TRIM21 protein expression in spinal cord tissues from EAE mice at 28 dpi (n = 5). **E** Immunofluorescence co-localization analysis of TRIM21 (red) and the astrocyte marker GFAP (green) in brain and spinal cord sections from control and EAE mice at 28 dpi (n = 5). Scale bar, 50 µm

To probe the in vivo relevance of TRIM21, we mined public transcriptomes (GSE138614) and found elevated TRIM21 mRNA in MS brain (Fig. 5A), a result corroborated in our MS patients’ PBMCs by RT-qPCR (Fig. 5B). In the EAE model, TRIM21 protein showed concordant increases in both brain and spinal cord tissues following MOG₃₅-₅₅ immunization (Fig. 5C and D). Notably, immunofluorescence staining further showed substantially increased TRIM21 expression specifically within astrocytes of EAE mice compared to controls (Fig. 5E).

### Targeting astrocytic TRIM21 ameliorates EAE and suppresses RGMa

**Fig. 6:**
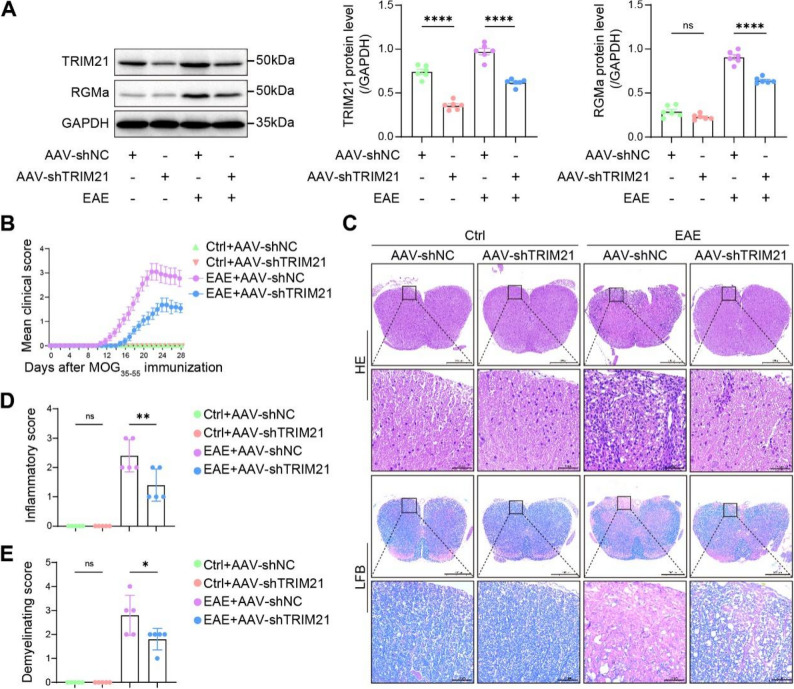
Intracerebroventricular delivery of TRIM21 knockdown ameliorates disease severity in the EAE model. **A** Following AAV-shTRIM21 infection, the expression of TRIM21 and RGMa was analyzed and quantified by immunoblotting to assess knockdown efficiency at 28 dpi (n = 6). **B** Clinical manifestation scores after AAV-shTRIM21 or AAV-shNC administration in control or EAE mice (n = 11). **C** Histological analysis of spinal cord sections (HE and LFB staining) from EAE and control mice post-AAV treatment (n = 5). **D** Inflammatory cell density quantified (n = 5). **E** Demyelination area quantified in spinal cord sections (n = 5)

Astrocyte-specific knockdown of TRIM21 was achieved using an AAV9 vector under the control of the gfaABC1D promoter to investigate the role of TRIM21 in EAE. Four weeks prior to MOG_35−55_ immunization, mice received an intracerebroventricular injection of AAV-shTRIM21 to achieve astrocyte-specific TRIM21 knockdown (Fig. S6A). Enhanced green fluorescent protein (EGFP) immunoreactivity co-localized exclusively with GFAP, but not with neuron-specific nuclear protein (NeuN) or ionized calcium-binding adapter molecule 1 (Iba1), confirming astrocytic targeting (Fig. S6B). Western blot analysis further validated the significant reduction of TRIM21 protein in treated mice. Consistently, RGMa protein levels were also downregulated in the EAE model following TRIM21 knockdown (Fig. 6A). Astrocyte-specific TRIM21 knockdown resulted in attenuated EAE severity during the acute phase, as reflected by improved clinical scores (Fig. 6B). Histopathological evaluation via HE and LFB staining revealed a substantial reduction in inflammation and demyelination in the spinal cords of these mice (Fig. 6C-E).

### Astrocyte-specific targeting of TRIM21 alleviates EAE by regulating the RGMa and CCL2

**Fig. 7:**
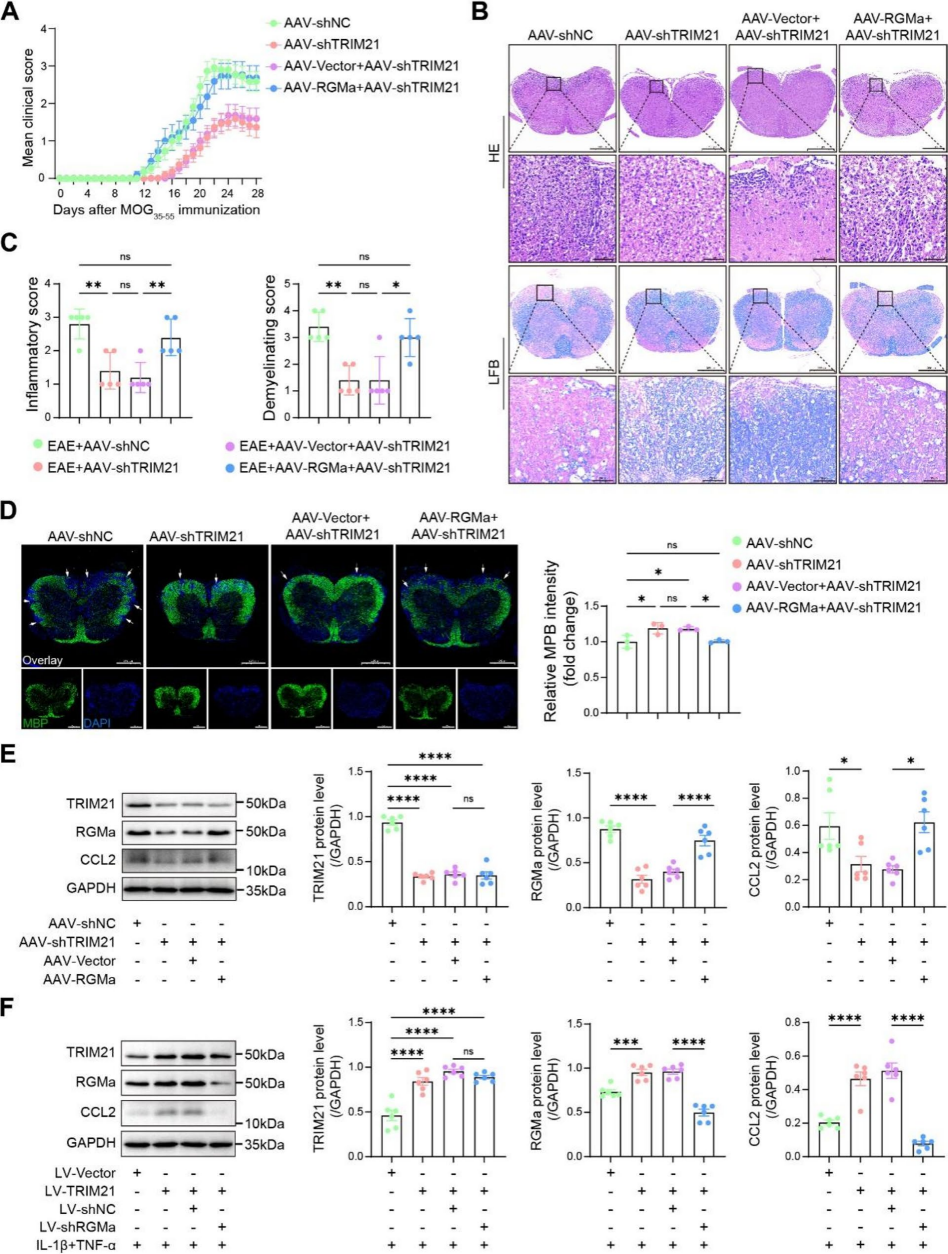
TRIM21 knockdown alleviates EAE symptoms and astrocytic activation, whereas RGMa overexpression reverses these benefits. **A** Clinical scores of EAE mice following AAV-mediated TRIM21 knockdown and/or RGMa overexpression (n = 11). **B** Histology (HE and LFB staining) of spinal cords after modulation of TRIM21 and RGMa at 28 dpi (n = 5). **C** Quantification of inflammatory infiltration and demyelination in spinal cord tissues under indicated treatments (n = 5). **D** Immunofluorescence staining and quantification of MBP loss (n = 3). **E** Representative immunoblots and quantification of TRIM21, RGMa, and CCL2 expression levels in manipulated EAE mice (n = 6). **F** Representative immunoblots and quantification of corresponding protein levels following TRIM21 overexpression and/or RGMa knockdown in astrocytes (n = 6)

To delineate the role of astrocytic TRIM21 in regulating RGMa in EAE, we modulated the TRIM21-RGMa specifically in astrocytes using AAV9 vectors driven by the gfaABC1D promoter. Astrocyte-specific knockdown of TRIM21 improved neurologic deficits in EAE mice along with reducing inflammation and demyelination. Meanwhile, myelin basic protein loss was attenuated. Notably, concurrent overexpression of RGMa partially reversed the therapeutic benefits of TRIM21 knockdown, suggesting that RGMa operates downstream of TRIM21 to promote disease progression (Fig. 7A-D).

Given that CCL2 is a key chemokine responsible for monocyte/macrophage recruitment in EAE [30], and based on our prior finding that RGMa directly regulates CCL2 expression (Fig. 1I, Fig. S3), we hypothesized that TRIM21 controls neuroinflammation through an RGMa-CCL2 pathway. In vivo, astrocyte-specific TRIM21 knockdown reduced both RGMa and CCL2 protein levels in the spinal cord, whereas reintroducing RGMa restored CCL2 expression (Fig. 7E). LV-TRIM21 in cytokine-activated astrocytes upregulated RGMa and CCL2 in vitro, while RGMa silencing reversed the effect (Fig. 7F). Together, these results established that TRIM21 propagates neuroinflammation in EAE by positively regulating the RGMa-CCL2 in astrocytes. As nuclear factor kappa-B (NF-κB) signaling is a major upstream regulator of CCL2 transcription, we examined whether the TRIM21-RGMa converged on this pathway. Western blot analysis showed that astrocyte-specific TRIM21 knockdown in EAE mouse spinal cords reduced phosphorylated p65 (p-p65) levels, which was reversed by RGMa overexpression (Fig. S7A). Consistently, overexpression of TRIM21 in primary astrocytes increased p-p65 levels, and this effect was reversed by RGMa knockdown (Fig. S7B). These data suggest that TRIM21-mediated RGMa stabilization may promote neuroinflammation, at least in part, through NF-κB activation.

### Amelioration of EAE by the HDACi Quisinostat is associated with suppression of the TRIM21-mediated RGMa stabilization and subsequent downregulation of CCL2

**Fig. 8:**
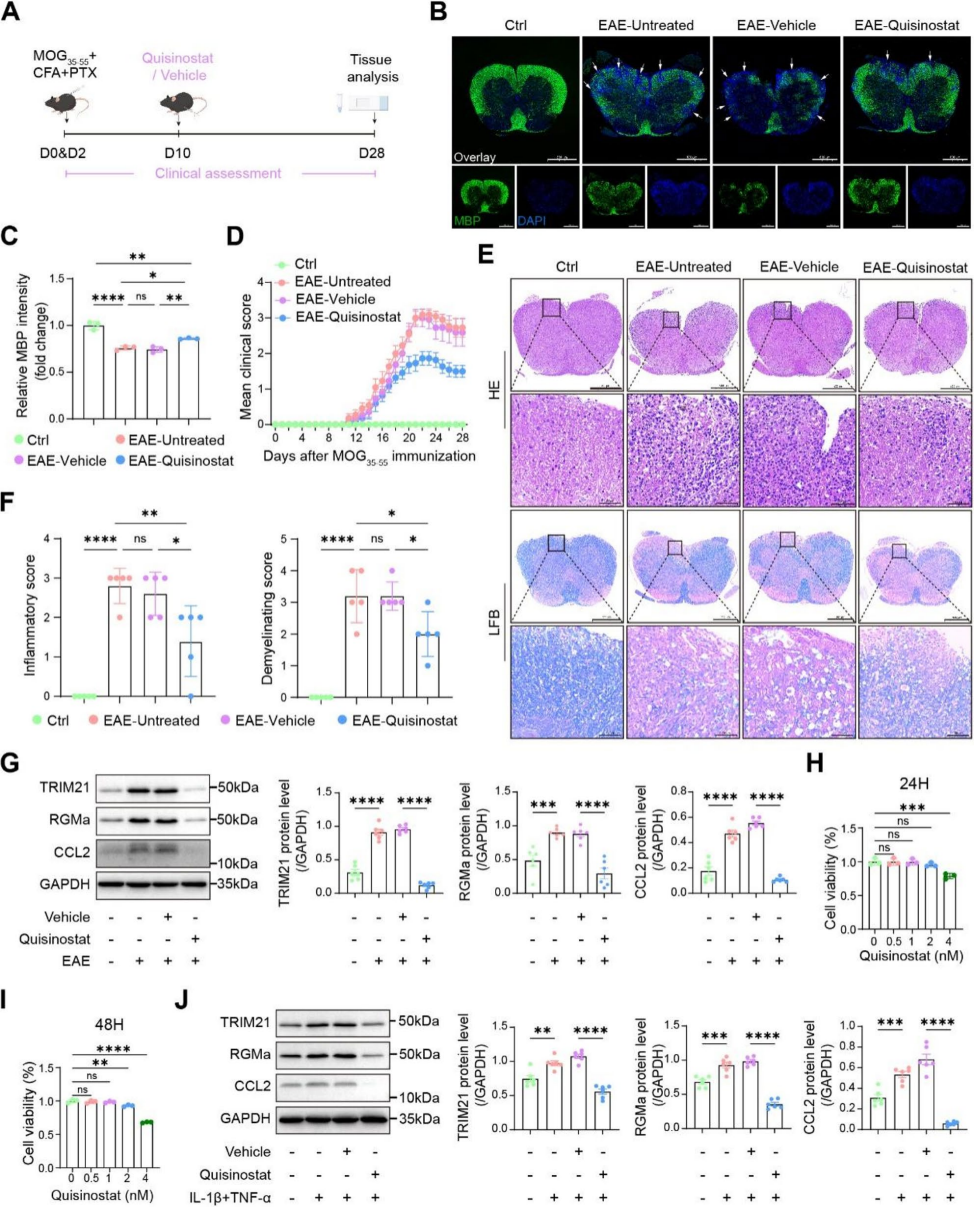
Quisinostat treatment ameliorates EAE pathology and modulates the TRIM21/RGMa/CCL2. **A** Schematic of the Quisinostat treatment regimen in the EAE mouse model. **B** and **C** Immunofluorescence analysis of MBP distribution in the spinal cord following Quisinostat intervention, indicating remyelination at 28 dpi (n = 3). **D** Following the administration of Quisinostat, clinical neurological scoring of EAE mice and control mice were recorded (n = 11). **E** and **F** Histopathological analyses of inflammatory cell infiltration and demyelination were carried out on tissues from Quisinostat-treated mice at 28 dpi (n = 5). **G**. Western blot analysis of spinal cord lysates showed altered protein expression of TRIM21, RGMa, and CCL2 after Quisinostat treatment at 28 dpi (n = 6). **H** and** I** Viability of primary astrocytes treated with Quisinostat was assessed by CCK-8 assay at 24 and 48 hours (n = 3). ** J** Proteins were extracted at 24 h post-treatment. In primary astrocytes, Quisinostat treatment in vitro correspondingly modulated the protein expression of TRIM21, RGMa, and CCL2 (n = 6)

Quisinostat, an HDACi previously shown to inhibit tumor progression and reduce TRIM21 protein levels in various cell types, has not been explored in autoimmune disease. We observed that Quisinostat treatment ameliorated EAE severity, diminished the areas of spinal cord inflammation and demyelination versus vehicle (Fig. 8A-F). At the molecular level, Western blot analysis suggested that Quisinostat concurrently downregulated protein expression of TRIM21, RGMa, and CCL2 in EAE mice (Fig. 8G). In astrocytes stimulated with IL-1β plus TNF-α for 24 h, 2 nM Quisinostat did not alter viability at 24–48 h (Fig. 8H, I), yet recapitulated the protein suppression observed in vivo (Fig. 8J). To assess whether Quisinostat acts through TRIM21, we overexpressed TRIM21 in astrocytes treated with Quisinostat. TRIM21 overexpression attenuated Quisinostat-induced downregulation of RGMa and CCL2 (Fig. S8), indicating that Quisinostat modulates these targets, at least in part, through TRIM21-dependent mechanisms.

## Discussion

**Fig. 9:**
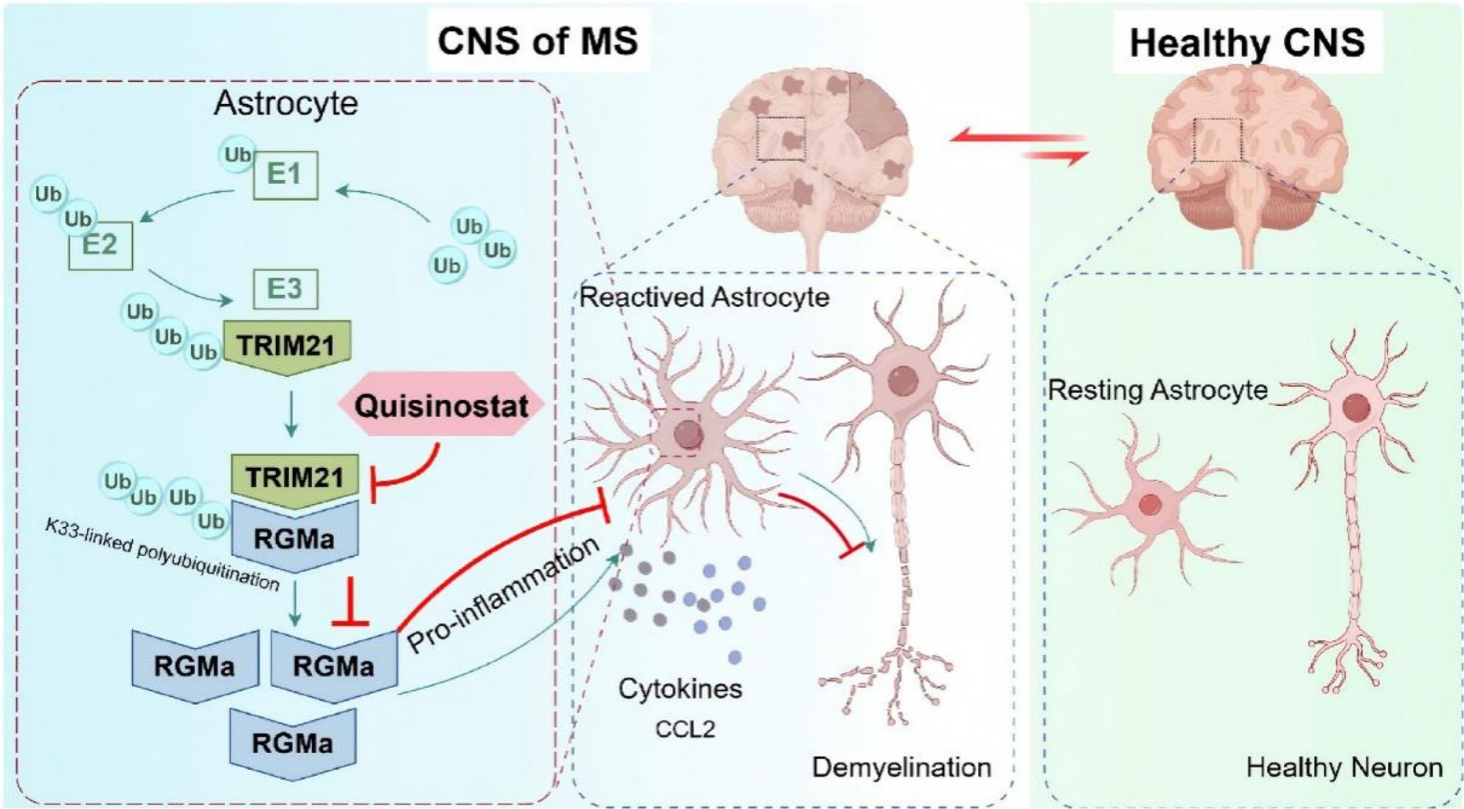
Schematic diagram of the interaction between TRIM21 and RGMa in reactive astrocytes of the CNS during EAE. TRIM21 binds to and stabilizes RGMa by promoting its K33-linked polyubiquitination, thereby enhancing reactive astrocyte activity and secretion of inflammatory factors (such as CCL2), which promotes inflammatory infiltration and demyelination

Here, we report that RGMa is highly expressed in MS patients, consistent with prior findings, and is enriched in astrocytes. We identify the E3 ubiquitin ligase TRIM21 as a regulator of RGMa protein stability. Mechanistically, the B30.2/SPRY domain of TRIM21 binds to amino acid region 118–303 of RGMa, catalyzing K33-linked polyubiquitylation at K238 of RGMa and stabilizing RGMa in cellular models. This amplifies the RGMa/CCL2 signal, driving astrocytic activation and the release of pro-inflammatory mediators. Furthermore, we demonstrate that Quisinostat alleviates EAE pathology during the acute phase (Fig. 9).

Accumulating evidence indicates that RGMa exhibits broad expression across diverse neural and immune cell populations within the central nervous system. In MS lesions and EAE, RGMa is detected in CD68^+^ macrophages/microglia, CD11b^+^ microglia, CD45^+^ leukocytes, CD4^+^ T cells (particularly Th17 subsets), and bone marrow-derived dendritic cells, where it actively promotes neuroinflammation and T cell-mediated immune responses [12, 31–33]. Additionally, endothelial RGMa contributes to blood-brain barrier disruption [13], while oligodendrocyte RGMa may impair remyelination [33]. This multicellular expression pattern suggests that RGMa operates through distinct, cell-type-specific mechanisms to coordinate pathology in MS/EAE.

However, to the best of our knowledge, no previous studies have specifically characterized RGMa expression in neurons and astrocytes within MS/EAE contexts. Intriguingly, in neuromyelitis optica spectrum disorder (NMOSD), another neuroinflammatory disease, RGMa is expressed by spared neurons and astrocytes, where it drives neutrophil-associated astrocytopathy. This suggests that resident neural cells represent an underappreciated source of RGMa in neuroinflammatory diseases. Indeed, RGMa has been increasingly implicated as a critical regulator in various neurological conditions, including multiple sclerosis [34, 35]. While previous work demonstrated that astrocytic RGMa expression is transcriptionally induced by TGFβ1/Smad signaling in stroke models [36], the mechanisms governing RGMa protein stability in reactive astrocytes during neuroinflammation remained unclear. Our study identifies TRIM21-mediated K33-linked ubiquitination as a key post-translational mechanism stabilizing RGMa, thereby extending these previous observations and suggesting that protein stability regulation may serve as an additional regulatory node in astrocyte-driven neuroinflammation in EAE.

EAE is a complex autoimmune demyelinating disease involving the interplay of multiple cellular events, including peripheral immune cell infiltration, microglial activation, oligodendrocyte injury, and reactive astrogliosis [5, 37]. Among these, reactive astrocytes serve as critical orchestrators. They function not only as essential components of the blood-brain barrier (BBB) and supporters of neuronal function, but also as central hubs in neuroimmune communication [38, 39]. Upon activation, astrocytes actively recruit peripheral immune cells into the central nervous system through massive release of chemokines such as CCL2 and CXCL10. Simultaneously, their production of proinflammatory cytokines including IL-1β and TNF-α establishes positive feedback loops with microglia, jointly amplifying inflammatory cascades and directly or indirectly compromising myelin integrity and repair [40–43]. Therefore, elucidating the upstream mechanisms driving aberrant astrocytes activation is of particular importance for understanding the pathogenesis of MS/EAE and identifying potential intervention nodes.

We further reveal that modulating RGMa expression levels in astrocytes directly influences the production of key inflammatory factors, including IL-1β, IL-6, and CCL2. RGMa knockdown reduced multiple inflammatory factors, including CCL2. CCL2 is a key astrocyte-derived pro-inflammatory chemokine in MS. The aberrant release of inflammatory factors such as CCL2 triggers microglia to release myelin-damaging substances, while simultaneously inhibiting the differentiation of oligodendrocyte precursor cells, thereby impairing myelin repair and regeneration [30, 44]. These results suggest that RGMa contributes to astrocyte-mediated inflammation.

Ubiquitination, a critical post-translational modification, serves as a molecular switch that broadly regulates protein stability and activity [45, 46]. E3 ubiquitin ligases determine substrate specificity [47–49]. The TRIM family, a major class of E3 ligases, plays central roles in various immune-mediated diseases, including inflammatory bowel disease and systemic lupus erythematosus (SLE) [50–52]. In this study, we identify TRIM21 as a previously unrecognized E3 ligase for RGMa using IP-MS. Ubiquitin chains can be formed via any of seven lysine residues (K6, K11, K27, K29, K33, K48, K63) or the N-terminal methionine (M1) [53, 54]. K48- and K63-linked chains are the most prevalent, classically associated with proteasomal degradation and signal transduction, respectively [55, 56]. In contrast to K63-linked ubiquitination of PKM2 driving metabolic reprogramming [18], our results indicate that TRIM21 stabilizes RGMa in inflammatory contexts via K33-linked ubiquitination, a non-canonical modification confirmed in cell culture systems. Unlike canonical K48-linked degradation or K63-linked signaling, K33-linked polyubiquitination has emerged as a non-degradative modification regulating protein stability and intracellular trafficking [54]. Similar to the stabilization of TANK-binding kinase 1 by K33-linked chains [57] and that of NLRP3 [58], our data suggest that TRIM21-mediated K33 ubiquitination may protect RGMa from proteasomal degradation. Furthermore, given that K33 ubiquitination regulates protein trafficking from the Golgi apparatus [59], it is plausible that this modification may also influence RGMa subcellular localization in astrocytes, although this remains to be determined.

We delineate a direct interaction between the B30.2/SPRY domain of TRIM21 and amino acids 118–303 of RGMa and pinpoint K238 of RGMa as the critical residue modified by K33-linked ubiquitination. The B30.2/SPRY domain of TRIM21 is known to mediate protein-protein interactions and is essential for targeting substrates for ubiquitination [60, 61]. Our work further defines the 118–303 region of RGMa as an interaction interface with an E3 ubiquitin ligase. Furthermore, the K238 site of RGMa is positioned within its TRIM21-binding region. To functionally validate the importance of K238, we performed cycloheximide chase assays comparing wild-type RGMa and the K238R mutant. The K238R mutant exhibited markedly accelerated protein degradation compared to wild-type RGMa, indicating that ubiquitination at this site is critical for TRIM21-mediated stabilization in vitro. These findings suggest that K33-linked ubiquitin chains at K238 protect RGMa from proteasomal degradation, providing a mechanistic explanation for RGMa accumulation during neuroinflammation. However, we recognize that these experiments were conducted in cultured cells, and further studies in animal models carrying the RGMa-K238R mutation will be needed to confirm the role of this site in MS/EAE pathogenesis.

TRIM21 has been implicated in various cellular processes [62–64] and has catalyzed K63-linked ubiquitination of p62/SQSTM1 exacerbating SLE [52]. This suggests that TRIM21 may engage substrates to modulate inflammation in autoimmune contexts. To define the contribution of TRIM21 to MS, we demonstrated its significant upregulation in MS patients and EAE, with prominent enrichment in astrocytes. Its astrocyte-specific knockdown alleviated EAE progression and reduced both RGMa and CCL2 levels in vivo; these effects were partially reversed by RGMa rescue. In contrast, TRIM21 overexpression increased CCL2 levels in vitro, which was blocked by RGMa knockdown. These results indicate a functional link between TRIM21 and RGMa in regulating CCL2. While our in vitro epistasis experiment supports RGMa functioning downstream of TRIM21, definitive in vivo validation through simultaneous cell-specific manipulations will be an important goal for future studies.

These results collectively suggest that TRIM21 promotes astrocyte-mediated neuroinflammation in EAE by stabilizing RGMa and thereby enhancing CCL2 expression. While this study establishes this molecular pathway, how RGMa mechanistically drives CCL2 transcription remains unclear. NF-κB is a known CCL2 regulator [65, 66]. Our additional data show that TRIM21 and RGMa knockdown or overexpression affects NF-κB activation in vivo and in vitro, suggesting that this pathway may link RGMa to CCL2 induction. However, we have not demonstrated direct interaction between RGMa and NF-κB components, and additional signaling pathways may contribute. Future studies are needed to determine whether RGMa regulates CCL2 transcription directly or through intermediate molecules. Certainly, the pathogenesis of EAE involves complex multicellular interactions. While the present work primarily elucidates the molecular mechanisms operating within astrocytes, whether TRIM21-RGMa signaling has similar functions in other disease-relevant cell types (such as microglia or infiltrating myeloid cells) and how this signal mediates intercellular communication represent important directions for future investigation.

Notably, as a typical E3 ubiquitin ligase, TRIM21 likely coordinates cellular responses through multiple substrates. Previous work showed that TRIM21 modulates astrocyte metabolism via PKM2 in EAE [18]. Our finding that TRIM21 amplifies inflammatory signaling by stabilizing RGMa in the autoimmune demyelinating setting reveals a distinct, yet potentially complementary, mechanism. We propose that TRIM21 may cooperatively drive astrocytes into a pro-inflammatory state through different substrates: reprogramming metabolism via PKM2 while amplifying inflammatory signals via RGMa. Although the full spectrum of TRIM21 substrates in reactive astrocytes remains to be elucidated, our work identifies RGMa as one of the key substrates responsible for its pro-inflammatory effects in EAE/MS. How these distinct substrates, including PKM2 and RGMa, coordinate to shape astrocyte responses during neuroinflammation remains to be fully elucidated.

Given the critical role of TRIM21, we treated EAE mice with Quisinostat, which is capable of reducing TRIM21 [20]. Quisinostat treatment alleviated inflammatory infiltration, demyelination, and neurological deficits in EAE mice, and reduced TRIM21, RGMa, and CCL2 protein levels. Notably, Quisinostat is a tolerable and brain-penetrant agent [19]. However, as a broad-spectrum HDACi, Quisinostat engages numerous targets [67, 68]. Rescue experiments in astrocytes showed that TRIM21 overexpression attenuates Quisinostat-induced downregulation of RGMa and CCL2, suggesting that Quisinostat acts, at least in part, through TRIM21. We recognize that these cell-based findings do not exclude contributions from other HDACi targets in vivo, and definitive confirmation of TRIM21’s role in Quisinostat’s therapeutic effects will require studies in animal models with astrocyte-specific TRIM21 deletion. Additionally, future studies should employ conditional interventions after clinical symptom onset in EAE to evaluate the therapeutic window and efficacy of modulating TRIM21-mediated RGMa stabilization.

However, several limitations warrant consideration and point to future directions. First, constrained by the accessibility of human CNS tissues, the correlation between the peripheral measures and their expression within the CNS lesions remains to be validated. Second, while our data demonstrate that NF-κB activation correlates with RGMa-dependent CCL2 induction, the direct mechanistic link remains to be fully elucidated. Third, the specific K33-linked ubiquitination of RGMa at K238 by TRIM21, while robustly demonstrated in cell-based systems, awaits direct causal confirmation in vivo. To establish this causality, future studies utilizing mice with astrocyte-specific TRIM21 overexpression combined with RGMa K238R knock-in (to block ubiquitination at this site), as well as astrocyte-specific TRIM21 knockout using Tg(GFAP-cre)8Gtm mice (which achieves complete gene deletion), will be necessary to determine whether this modification drives disease progression during both acute and chronic phases of EAE, thereby evaluating its potential as a therapeutic target at advanced disease stages. Fourth, while we observed RGMa signal near the nucleus, quantitative analysis of its subcellular distribution was not performed. Consequently, whether localization changes under inflammatory conditions or TRIM21 modulation remains to be determined in future studies employing orthogonal approaches such as live-cell confocal imaging, subcellular fractionation, and nuclear-cytoplasmic separation. Finally, future studies employing cell-specific genetic tools, such as Cx3cr1-CreER and Cd4-Cre mice, would help clarify the cellular localization of key proteins like RGMa and TRIM21, as well as formally dissect the drug’s cell-type-specific contributions. Furthermore, future work utilizing astrocyte-specific TRIM21-overexpressing mice with RGMa K238R knock-in will determine whether this signal sustains disease progression during acute and chronic phases, thereby establishing its potential as a therapeutic target at advanced disease stages.

In summary, this study demonstrates that TRIM21 (via its B30.2/SPRY domain) interacts with RGMa (amino acids 118–303) to catalyze K33-linked ubiquitination at K238 of RGMa and promote RGMa stability in cultured astrocytes and HEK293T cells. TRIM21-RGMa contributes to astrocyte activation and the release of inflammatory mediators such as CCL2 during the acute phase of EAE. These findings provide insights into the mechanistic understanding of EAE pathogenesis during the acute inflammatory phase and may offer a foundation for developing targeted therapies.

### Data Availability

No datasets were generated or analyzed during the current study.

## Supplementary Information

Supplementary Material 1.

Supplementary Material 2.

## Data Availability

No datasets were generated or analysed during the current study.

## Abbreviations

MS: Multiple sclerosis
RGMa: Repulsive guidance molecule a
EAE: Experimental autoimmune encephalomyelitis
CCL2: C–C motif chemokine ligand 2
TRIM21: Tripartite motif-containing(TRIM) protein 21
CNS: Central nervous system
UPS: Ubiquitin-proteasome system
PKM2: Pyruvate kinase M2
PBMCs: Peripheral-blood mononuclear cells
GEO: Gene Expression Omnibus
MOG_35–55_: Myelin oligodendrocyte glycoprotein peptide 35–55
PTX: Pertussis toxin
PBS: Phosphate buffer saline
HE: Hematoxylin and eosin
LFB: Luxol fast blue
RIPA: Radio-immunoprecipitation assay
Co-IP: Co-immunoprecipitation
IP-MS: Immunoprecipitation-mass spectrometry
RT-qPCR: Reverse-transcription quantitative real-time PCR
HDACi: Histone deacetylase inhibitor
ECL: Enhanced chemiluminescence
icv.: Intracerebroventricular
ip.: Intraperitoneally
dpi.: Days post immunization
PTMs: Post-translational modifications
CHX: Cycloheximide
SLE: Systemic lupus erythematosus
