# Supplementary material for: TRIM21 promotes astrocyte-mediated neuroinflammation in experimental autoimmune encephalomyelitis by stabilizing RGMa via K33-linked ubiquitination
Source: J Neuroinflammation. 2026 Mar 19;23:144. doi: 10.1186/s12974-026-03769-4 (PMC13123086; doi:10.1186/s12974-026-03769-4)
Supplement: Supplementary file 1 — Supplementary Material 1. [file 12974_2026_3769_MOESM1_ESM.docx]

**Supplementary Material**

# TRIM21 promotes astrocyte-mediated neuroinflammation in experimental autoimmune encephalomyelitis by stabilizing RGMa via K33-linked ubiquitination

Shaoru Zhang, Zijie Wang, Ruiqi Cheng, Xiaolin Yang, Anan Jiang, Li Zhu, Xinyue Qin


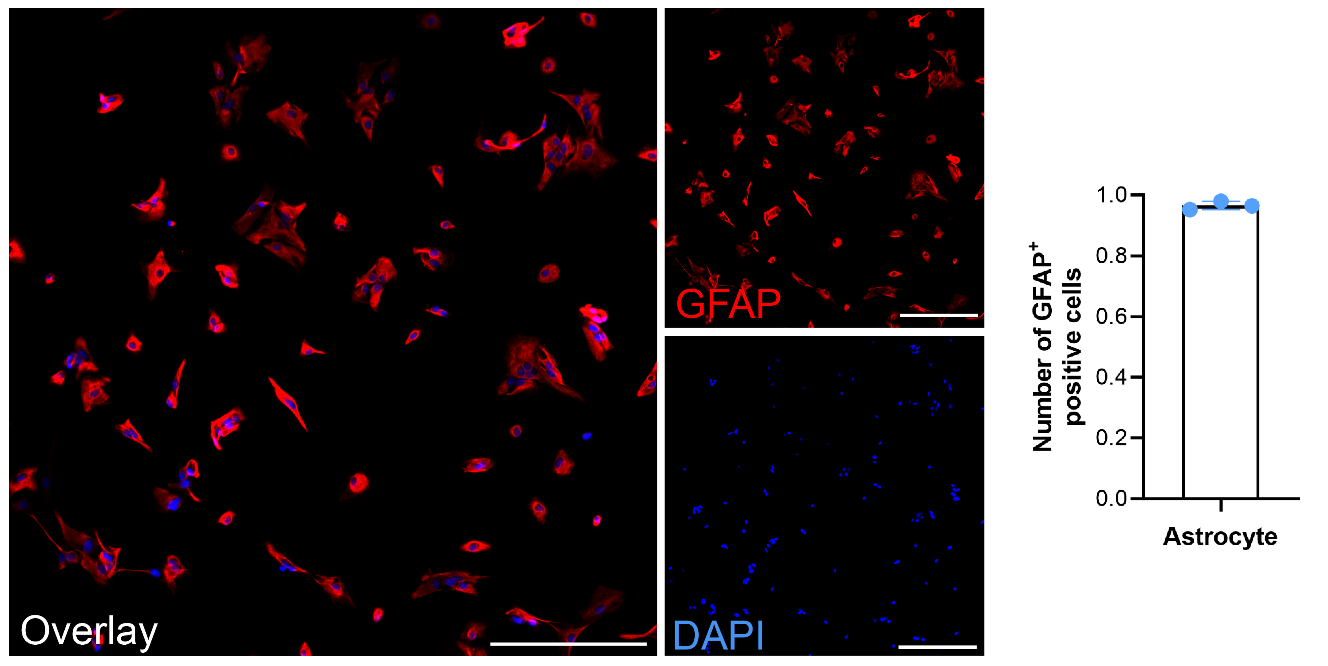


**Figure S1.** Characterization of primary astrocyte cultures. Representative immunofluorescence images showing GFAP (red, astrocyte marker) and DAPI (blue, nuclei) staining of purified primary cortical astrocytes. Scale bar = 311μm.


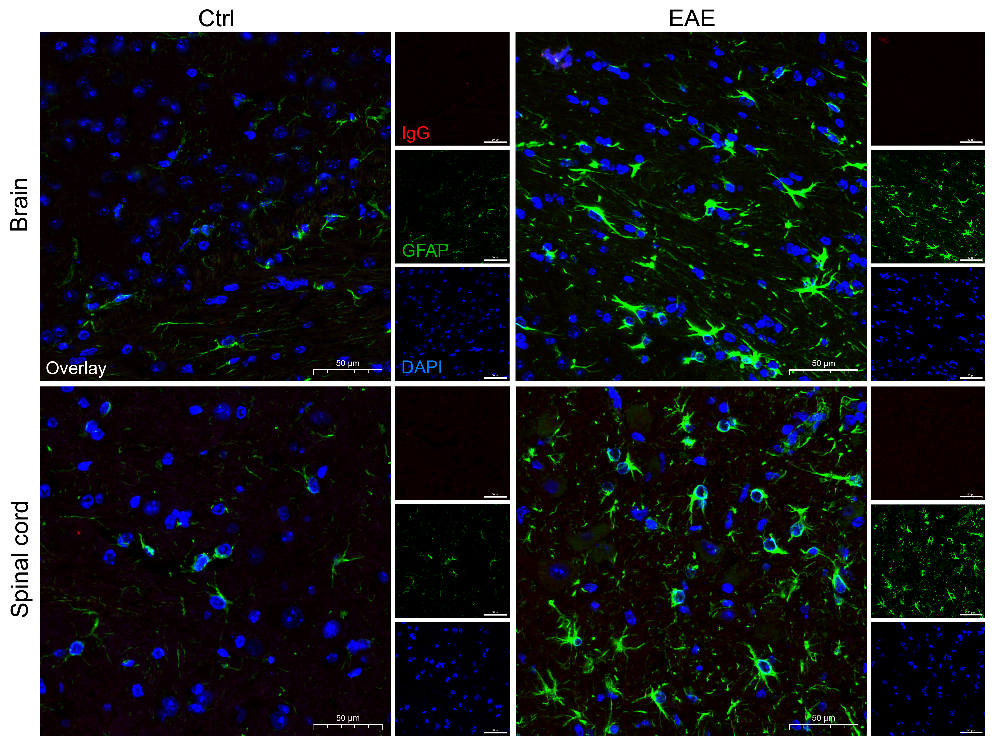


**Figure S2.** Negative control staining for RGMa immunofluorescence. Representative immunofluorescence images showing isotype control IgG (red) and GFAP (green) staining in brain and spinal cord sections from Control/EAE mice. Note the absence of nuclear IgG signal, confirming the specificity of the RGMa nuclear staining observed in Fig. 1H. Scale bar = 50μm.


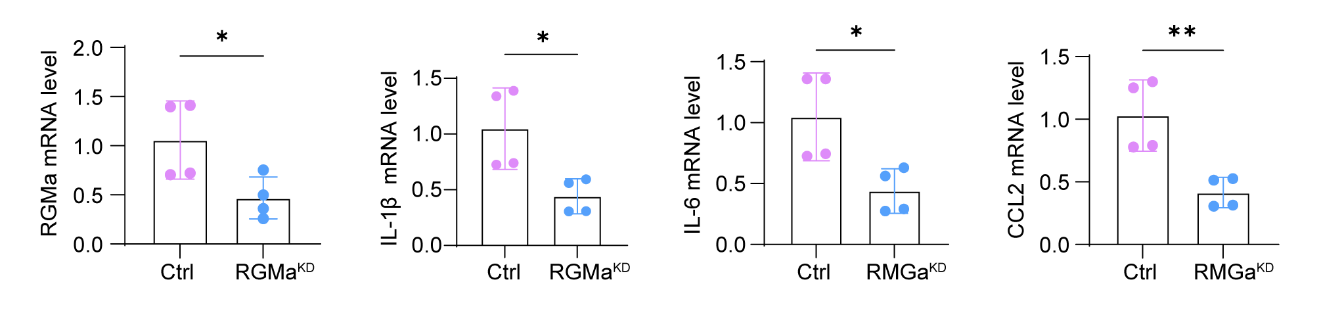


**Figure S3.** Inflammatory cytokine mRNA levels (IL-1α, IL-6, and CCL2) in primary astrocytes following lentivirus-mediated RGMa knockdown.


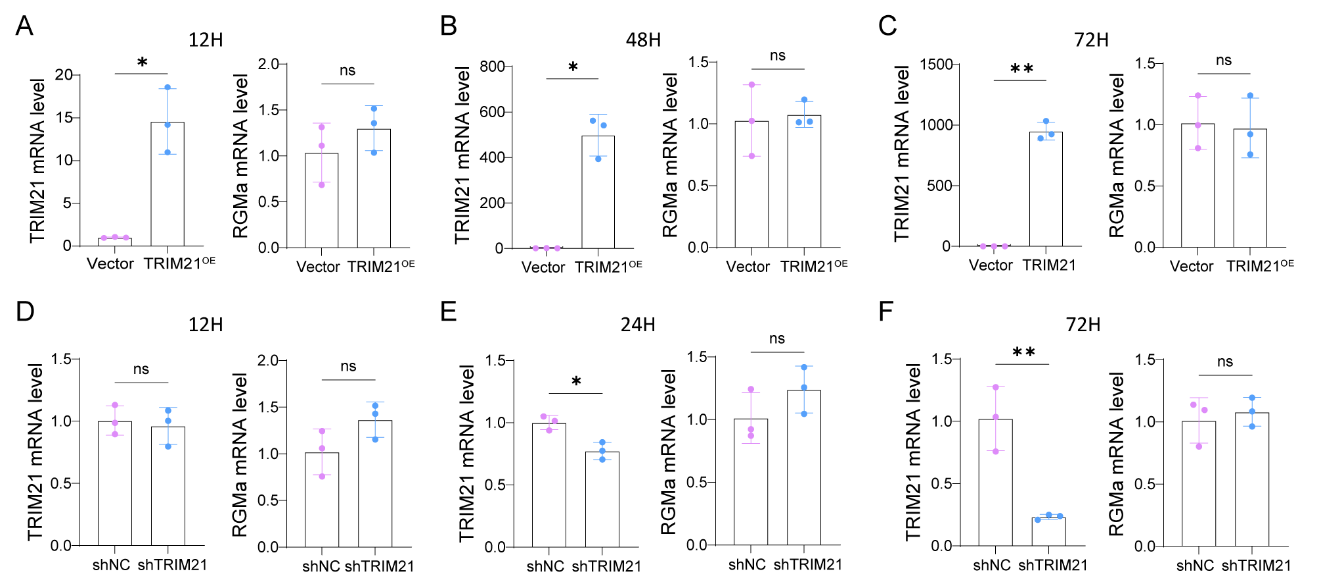


**Figure S4.** RT-qPCR analysis of RGMa mRNA levels in primary astrocytes at indicated time points following (A-C) TRIM21 overexpression (12, 48 and 72 h) or (D-F) TRIM21 knockdown (12, 24 and 72 h).


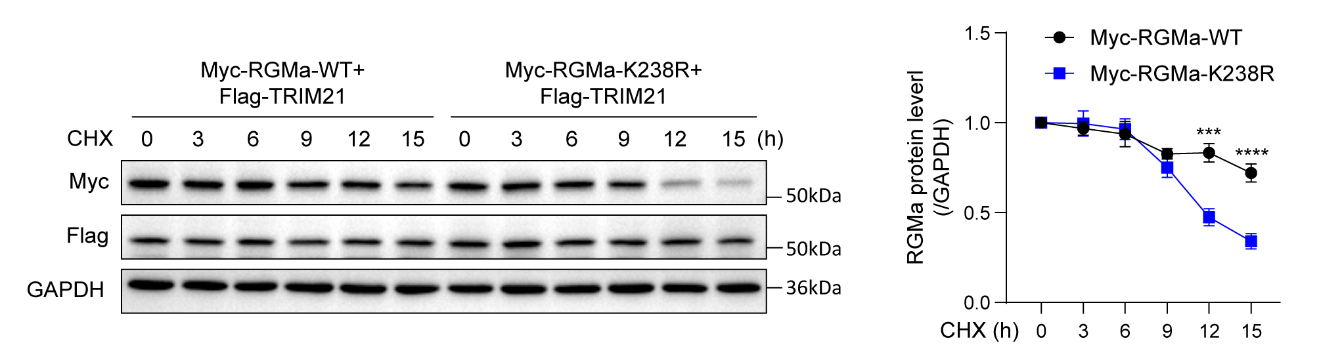


**Figure S5.** Representative western blot showing Myc-RGMa (WT or K238R mutant) protein levels at indicated time points after cycloheximide (CHX, 50 μg/ml) treatment in HEK293T cells co-expressing Flag-TRIM21.


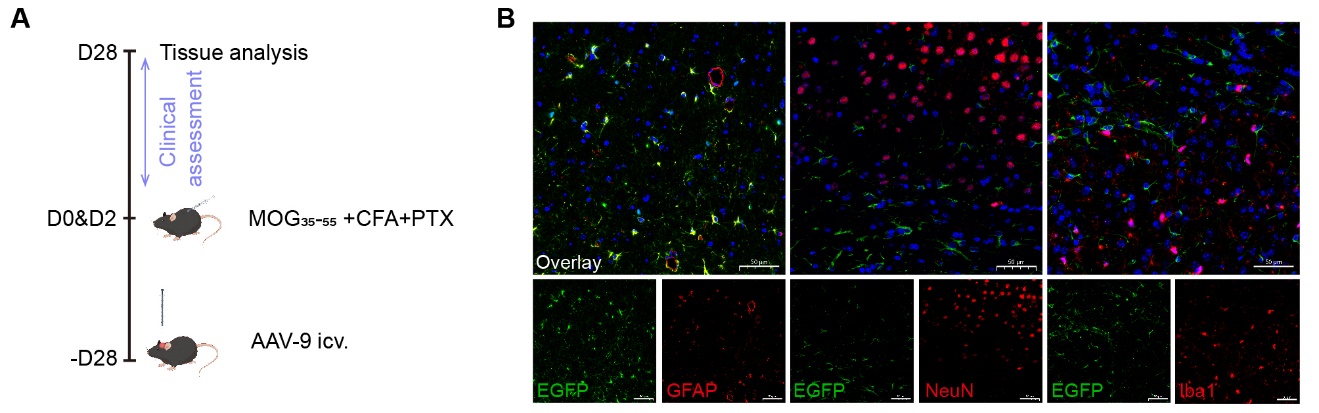


**Figure S6. Timeline of AAV9 delivery and immunization, and its specific tropism for astrocytes.**

(A) Timeline of intracerebroventricular AAV9 delivery and MOG_35-55_ immunization.

(B) Co-immunofluorescence analysis of EGFP and GFAP or NeuN or IBA1 of mice brain. Scale bar = 50μm.


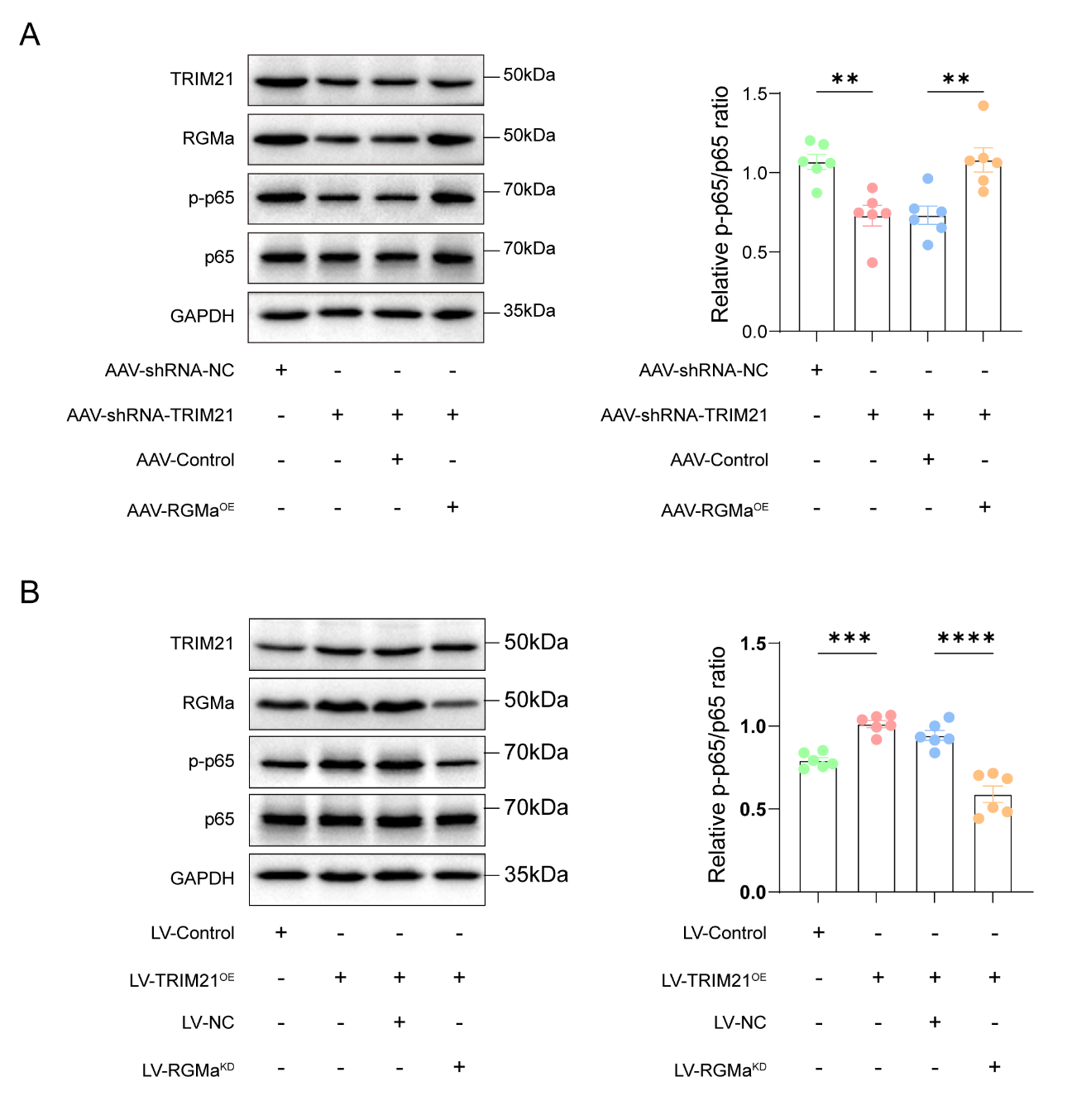


**Figure S7. The TRIM21-RGMa axis activates NF-κB signaling in EAE and primary astrocytes.**

(A) Representative western blot analysis of phosphorylated p65 (p-p65), total p65, TRIM21, and RGMa from EAE mice following astrocyte-specific TRIM21 knockdown (AAV-shTRIM21) and/or RGMa overexpression.

(B) Representative western blot showing p-p65, total p65, TRIM21, and RGMa in cytokine-stimulated primary astrocytes with TRIM21 overexpression and/or RGMa knockdown.
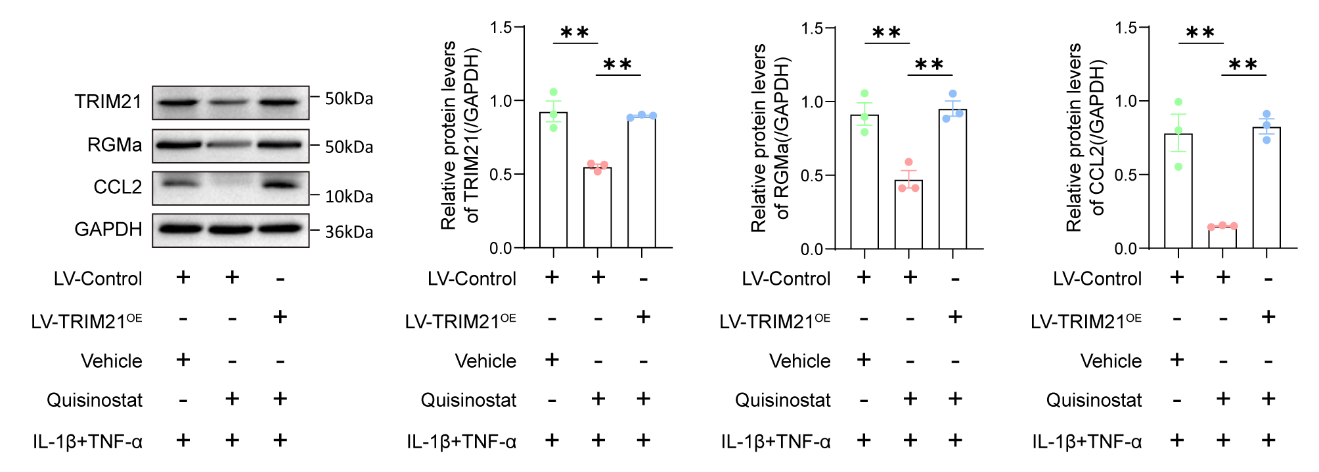


**Figure S8.** Representative western blot showing TRIM21, RGMa, and CCL2 levels in astrocytes stimulated with IL-1β plus TNF-α and treated with Quisinostat, with or without TRIM21 overexpression.

**Table S1. Primers used for gene cloning (5’-3’).**

| **Name** | **Primer sequences** |
| --- | --- |
| Human RGMa | Forward: TCCACTAGTCCAGTGTGGTGGAATTCGCCACCATGGGTGGCCTGGGGCCACGA |
|  | Reverse: AATCTGGTACGTCGTATGGGTAGCAGAACACAGGGAGCAGGGC |
| Human RGMa-ΔRegion1 | Forward: TCCACTAGTCCAGTGTGGTGGAATTCGCCACCATGAGCCAGCACAACTGCTCC |
|  | Reverse: AATCTGGTACGTCGTATGGGTAGCAGAACACAGGGAGCAGGGC |
| Human RGMa-ΔRegion2 | Forward-1: TCCACTAGTCCAGTGTGGTGGAATTCGCCACCATGGGTGGCCTGGGGCCACGA |
|  | Reverse-1: CTTCCTCTGGCATGAGGTCCTCTATGCCATGGACGGC |
|  | Forward-2: GGACCTCATGCCAGAGGAAGTGGTCAATGC |
|  | Reverse-2: AATCTGGTACGTCGTATGGGTAGCAGAACACAGGGAGCAGGGC |
| Human RGMa-ΔRegion3 | Forward: TCCACTAGTCCAGTGTGGTGGAATTCGCCACCATGGGTGGCCTGGGGCCACGA |
|  | Reverse: AATCTGGTACGTCGTATGGGTAGCGGACGGCAAAGGTCAGGTAG |
| Human TRIM21 | Forward: TTGGTACCGAGCTCGGATCCGCCACCATGGCTTCAGCAGCACGCTTGA |
|  | Reverse: CACCGTCATGGTCTTTGTAGTCGGATCCATAGTCAGTGGATCCT |
| Human TRIM21-ΔRING | Forward-1: TTGGTACCGAGCTCGGATCCGCCACCATGGCTTCAGCAGCACGCTTGA |
|  | Reverse-1: GAAAGCGCTGTGTGACCTCCTCCCACATCA |
|  | Forward-2: GGAGGTCACACAGCGCTTTCTGCTCAAGAA |
|  | Reverse-2: CACCGTCATGGTCTTTGTAGTCGGATCCATAGTCAGTGGATCCT |
| Human TRIM21-ΔB box | Forward-1: TTGGTACCGAGCTCGGATCCGCCACCATGGCTTCAGCAGCACGCTTGA |
|  | Reverse-1:GGACCATGGCCCGTTCCCCCTGTGTGCC |
|  | Forward-2: GGGGGAACGGGCCATGGTCCCTCTTGAGGA |
|  | Reverse-2: CACCGTCATGGTCTTTGTAGTCGGATCCATAGTCAGTGGATCCT |
| Human TRIM21-ΔCoiled coil | Forward-1: TTGGTACCGAGCTCGGATCCGCCACCATGGCTTCAGCAGCACGCTTGA |
|  | Reverse-1:CCAGTGCTGAAGGGACCATGGCGTGGTCAC |
|  | Forward-2: CATGGTCCCTTCAGCACTGGAACTGCTGCA |
|  | Reverse-2: CACCGTCATGGTCTTTGTAGTCGGATCCATAGTCAGTGGATCCT |
| Human TRIM21-ΔB30.2/SPRY | Forward-1: TTGGTACCGAGCTCGGATCCGCCACCATGGCTTCAGCAGCACGCTTGA |
|  | Reverse-1:ATCCAATATTTGGAGAGGTAATATCCAGGTCCT |
|  | Forward-2: TACCTCTCCAAATATTGGATCACAAGGATCCAC |
|  | Reverse-2: CACCGTCATGGTCTTTGTAGTCGGATCCATAGTCAGTGGATCCT |
| Human Ubiquitin-K6R | Forward: ATGCAGATTTTCGTGAGAACCCTTACCGGC |
|  | Reverse: GCCGGTAAGGGTTCTCACGAAAATCTGCAT |
| Human Ubiquitin-K11R | Forward: AAAACCCTTACCGGCAGGACCATCACCCTT |
|  | Reverse: AAGGGTGATGGTCCTGCCGGTAAGGGTTTT |
| Human Ubiquitin-K27R | Forward: CCATCGAAAATGTGAGGGCCAAGATCCAGG |
|  | Reverse: CCTGGATCTTGGCCCTCACATTTTCGATGG |
| Human Ubiquitin-K29R | Forward: TGTGAAGGCCAGGATCCAGGATAAGGAAGG |
|  | Reverse: CCTTCCTTATCCTGGATCCTGGCCTTCACA |
| Human Ubiquitin-K33R | Forward: CCAAGATCCAGGATAGGGAAGGCATTCCCC |
|  | Reverse: GGGGAATGCCTTCCCTATCCTGGATCTTGG |
| Human Ubiquitin-K48R | Forward: TCATCTTTGCAGGCAGGCAGCTGGAAGATG |
|  | Reverse: CATCTTCCAGCTGCCTGCCTGCAAAGATGA |
| Human Ubiquitin-K63R | Forward: TACAACATCCAGAGAGAGTCGACCCTGCAC |
|  | Reverse: GTGCAGGGTCGACTCTCTCTGGATGTTGTA |
| Human RGMa-K125R | Forward: AACTGCTCCCGGGATGGCCCCACCTCGCAG |
|  | Reverse: GGGGCCATCCCGGGAGCAGTTGTGCTGGCT |
| Human RGMa-K190R | Forward: CCAGACCTGCcgGGTGCAGGGCGCCTGGCC |
|  | Reverse: CGCCCTGCACCcgGCAGGTCTGGAAGCGGT |
| Human RGMa-K229R | Forward: CATCATCTTCcgGAACTTCCAGGAGTGTGT |
|  | Reverse: CCTGGAAGTTCcgGAAGATGATGGTGAGCT |
| Human RGMa-K238R | Forward: TGTGGACCAGCGGGTGTACCAGGCTGAGAT |
|  | Reverse: CTGGTACACCCGCTGGTCCACACACTCCTG |
| Human RGMa-K272R | Forward: GATCACTGAGCGGGTGTCAGGCCAGCACGT |
|  | Reverse: GCCTGACACCCGCTCAGTGATCTTCAGGCT |
| Human RGMa-K283R | Forward: GATCCAGGCCcgGTACATCGGCACCACCAT |
|  | Reverse: TGCCGATGTACcgGGCCTGGATCTCCACGT |
| shRNA-NC | GGTTCTCCGAACGTGTCACGT |
| shRNA-TRIM21(human) | GAGTTGGCTGAGAAGTTGGAA |

**Table S2. Antibodies used for co-immunoprecipitation analysis.**

| **Antibody target** | **Supplier** | **Catalog No.** |
| --- | --- | --- |
| Mouse anti-HA | Proteintech | 66006-2-Ig |
| Rabbit anti-Myc | Proteintech | 16286-1-AP |
| Mouse anti-Flag | Proteintech | 66008-4-Ig |
| Anti-mouse IgG | Proteintech | B900620 |
| Anti-rabbit IgG | Proteintech | 30000-0-AP |
| Mouse anti-RGMa | Santa Cruz Biotechnology | sc-393046 |

**Table S3. Primers used for RT-qPCR (5’-3’).**

| **Gene** | **Primers** sequences |
| --- | --- |
| Human RGMa | Forward: AACCAGCAGATCGACTTCCAG |
|  | Reverse: ACGGCTGTCTCGTATGGGA |
| Human TRIM21 | Forward: TCAGCAGCACGCTTGACAAT |
|  | Reverse: GGCCACACTCGATGCTCAC |
| Human β-Actin | Forward: CATGTACGTTGCTATCCAGGC |
|  | Reverse: CTCCTTAATGTCACGCACGAT |
| Human GAPDH | Forward: ACAACTTTGGTATCGTGGAAGG |
|  | Reverse: GCCATCACGCCACAGTTTC |
| Mouse RGMa | Forward: CCACACCTCAGGACTTTCACA |
|  | Reverse: AGACGGAAGTTCGTCCATTTC |
| Mouse TRIM21 | Forward: GGGAGGAGGTCACCTGTTCTA |
|  | Reverse: GGCACTCGGGACATGAACTG |
| Mouse Il1b | Forward: TGCCACCTTTTGACAGTGATG |
|  | Reverse: AAGGTCCACGGGAAAGACAC |
| Mouse Il6 | Forward: CGGCCTTCCCTACTTCACAA |
|  | Reverse: TTGCCATTGCACAACTCTTTTC |
| Mouse Ccl2 | Forward: ACCTGCTGCTACTCATTCACC |
|  | Reverse: CTCTTGAGCTTGGTGACAAAAACTA |
| Mouse β-Actin | Forward: GGCTGTATTCCCCTCCATCG |
|  | Reverse: CCAGTTGGTAACAATGCCATGT |
| Mouse GAPDH | Forward: AGGTCGGTGTGAACGGATTTG |
|  | Reverse: TGTAGACCATGTAGTTGAGGTCA |

**Table S4. Antibodies used for Western blot analysis.**

| **Antibody target** | **Supplier** | **Catalog No.** |
| --- | --- | --- |
| Rabbit anti-RGMa | Abcam | ab307652 |
| Rabbit anti-TRIM21 | Abcam | ab207728 |
| Rabbit anti-CCL2 | Affinity | DF7577 |
| Mouse anti-Flag | Proteintech | 66008-4-Ig |
| Rabbit anti-Flag | Proteintech | 20543-1-AP |
| Mouse anti-Myc | Proteintech | 60003-2-Ig |
| Mouse anti-HA | ABclonal | AE008 |
| Mouse anti-GAPDH | Proteintech | 60004-1-Ig |
| HRP-conjugated Goat Anti-Mouse IgG (H+L) | Proteintech | SA00001-1 |
| HRP-conjugated Goat Anti-Rabbit IgG (H+L) | Proteintech | SA00001-2 |

**Table S5. Antibodies used for immunofluorescence staining.**

| **Antibody target** | **Supplier** | **Catalog No.** |
| --- | --- | --- |
| Rabbit anti-RGMa | Proteintech | 12387-1-AP |
| Rabbit anti-TRIM21 | Proteintech | 12108-1-AP |
| Rabbit anti-MBP | Proteintech | 10458-1-AP |
| Mouse anti-GFP | Proteintech | 66002-1-Ig |
| Rabbit anti-GFAP | Proteintech | 16825-1-AP |
| Mouse anti-GFP | Proteintech | 66002-1-Ig |
| Rabbit anti-NeuN | Abcam | ab177487 |
| Rabbit anti-Iba1 | Abcam | ab178846 |
| Mouse anti-Flag | Proteintech | 66008-4-Ig |
| Rabbit anti-Myc | Proteintech | 16286-1-AP |
| Rabbit anti-IgG | Proteintech | 98136-1-RR |
| CoraLite488-conjugated Goat Anti-Mouse IgG(H+L) | Proteintech | SA00013-1 |
| CoraLite488-conjugated Goat Anti-Rabbit IgG(H+L) | Proteintech | SA00013-2 |
| CoraLite594-conjugated Goat Anti-Mouse IgG(H+L) | Proteintech | SA00013-3 |
| CoraLite594-conjugated Goat Anti-Rabbit IgG(H+L) | Proteintech | SA00013-4 |
