# Supplementary material for: TRIM21 promotes astrocyte-mediated neuroinflammation in experimental autoimmune encephalomyelitis by stabilizing RGMa via K33-linked ubiquitination
Source: J Neuroinflammation. 2026 Mar 19;23:144. doi: 10.1186/s12974-026-03769-4 (PMC13123086; doi:10.1186/s12974-026-03769-4)

Fig 1. D

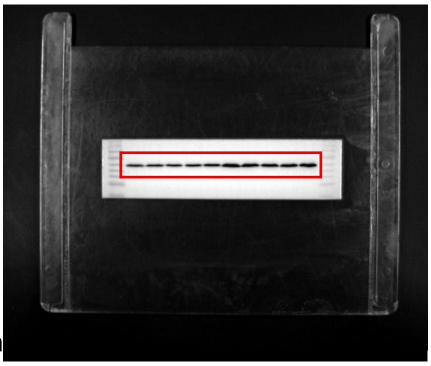

RGMa

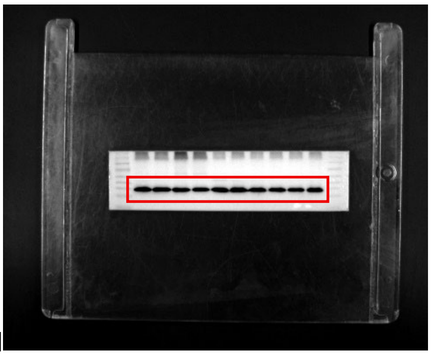

GAPDH

Fig 1. E

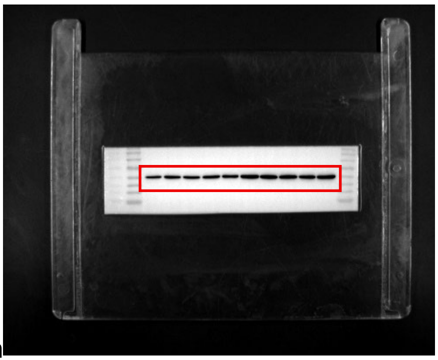

RGMa

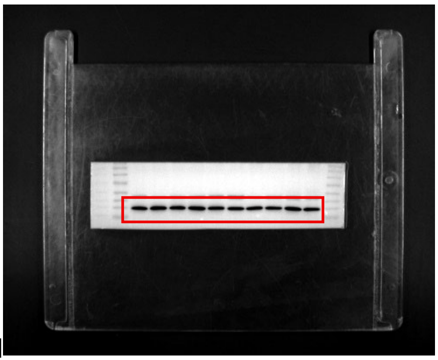

GAPDH

Fig 2. B

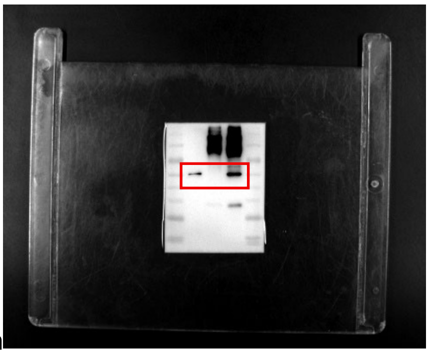

RGMa

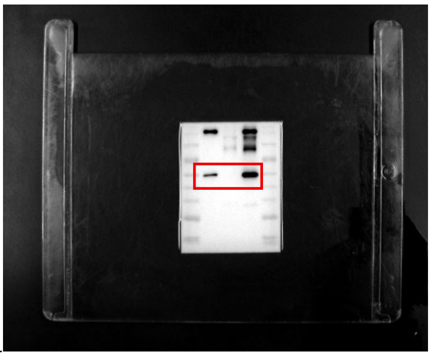

TRIM21

Fig 2. C

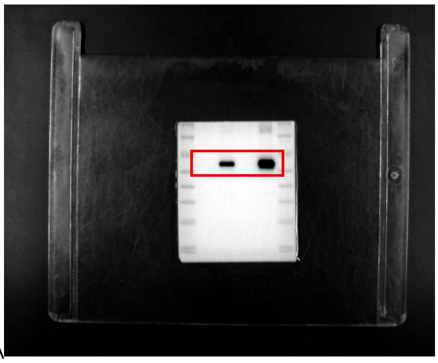

IB: HA

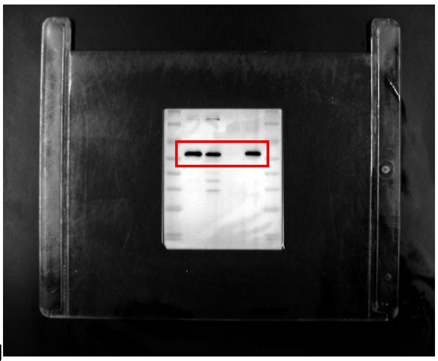

IB: Flag

Fig 2. D

IB: HA

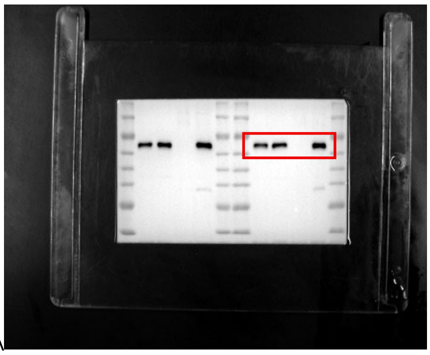

IB: Flag

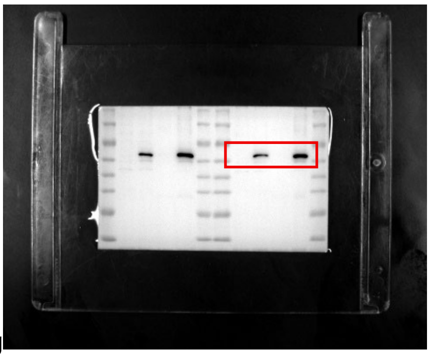

Fig 3. C

Myc

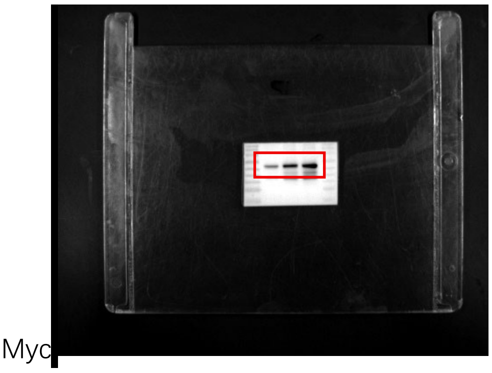

Flag

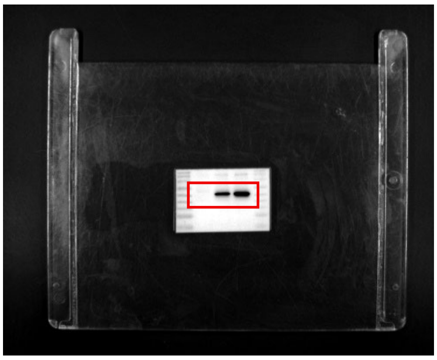

GAPDH

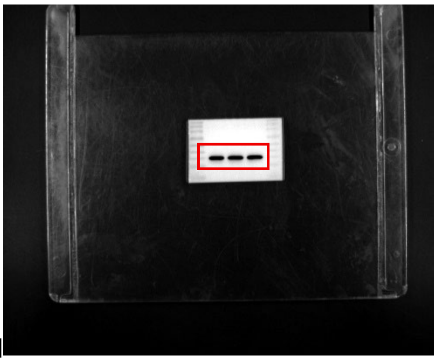

Fig 3. D

RGMa

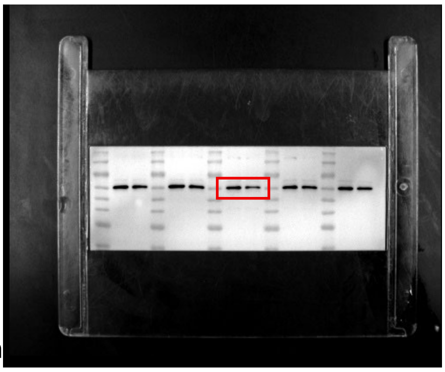

TRIM21

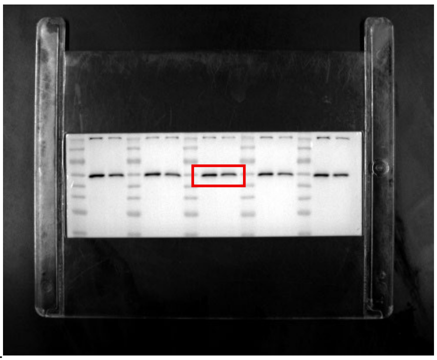

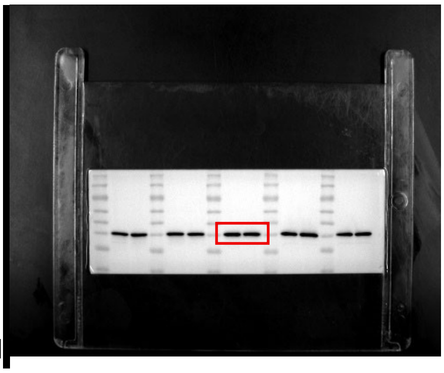

GAPDH

Fig 3. E

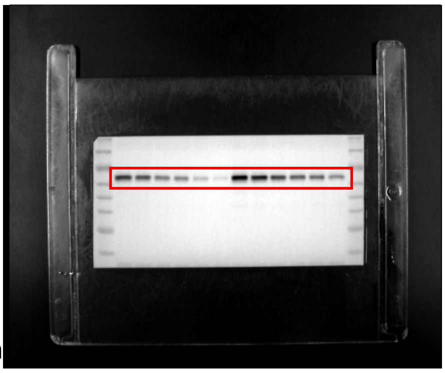

RGMa

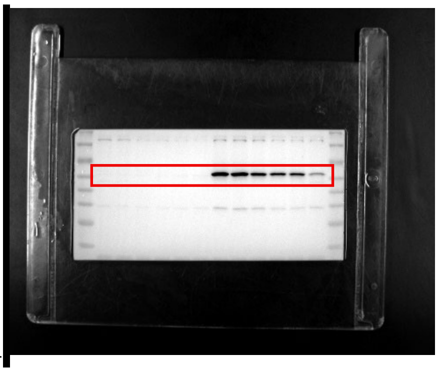

TRIM21

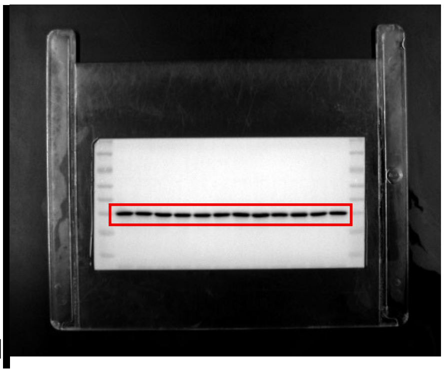

GAPDH

Fig 3. F

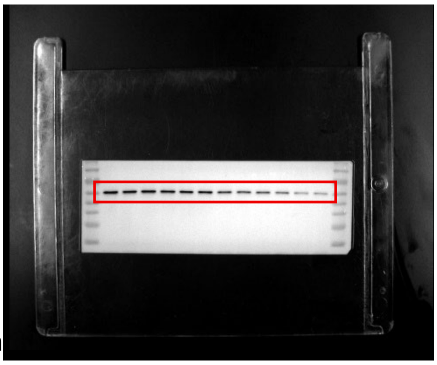

RGMa

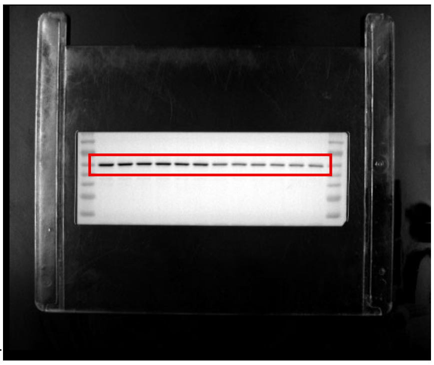

TRIM21

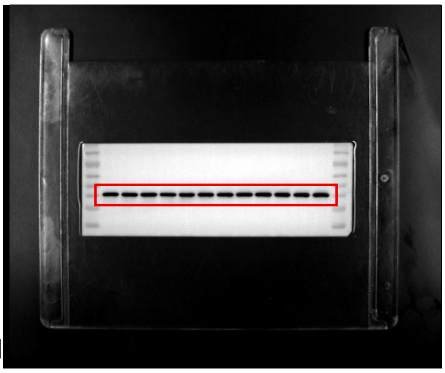

GAPDH

Fig 3. I

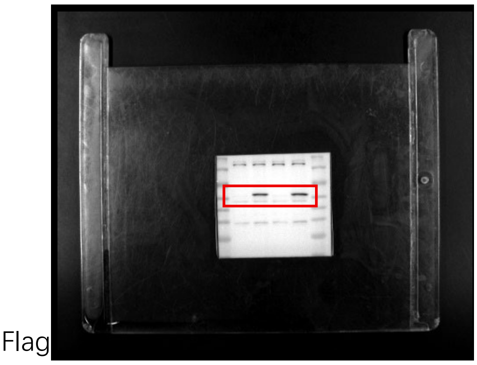

Flag

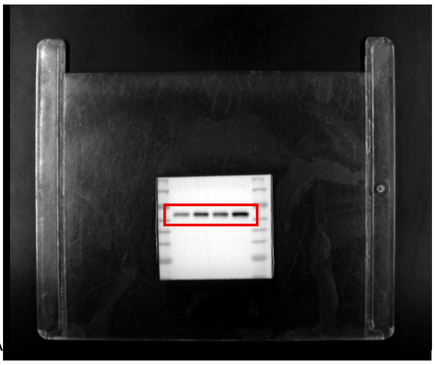

HA

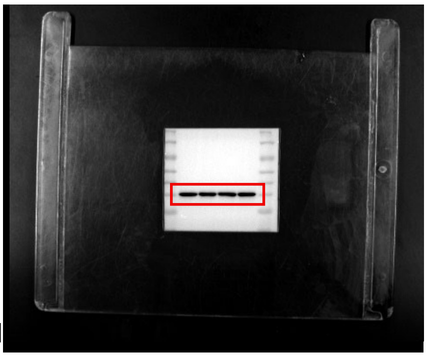

GAPDH

Fig 3. J

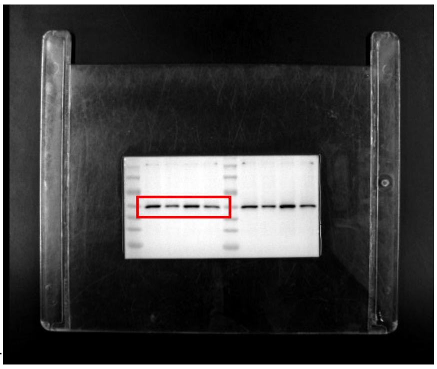

TRIM21

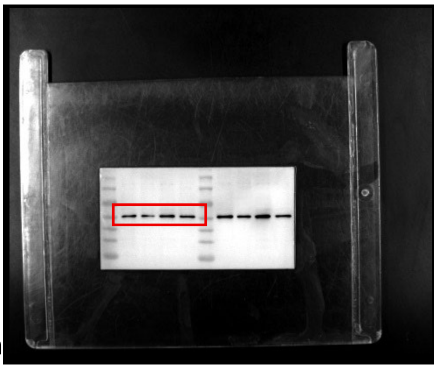

RGMa

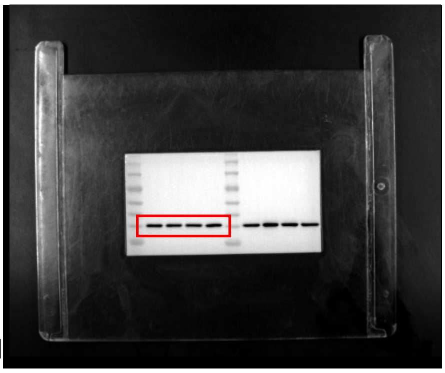

GAPDH

Fig4. A

IP

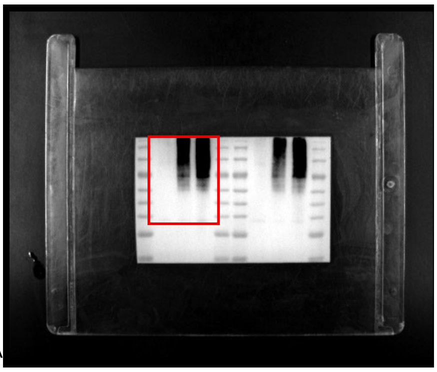

Blot: HA

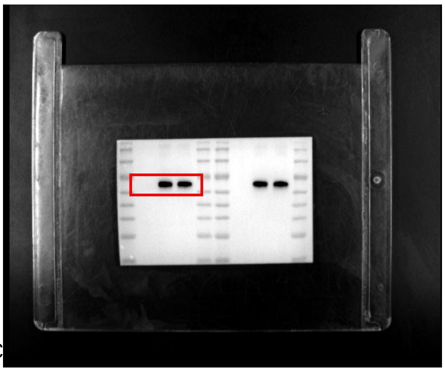

Blot: Myc

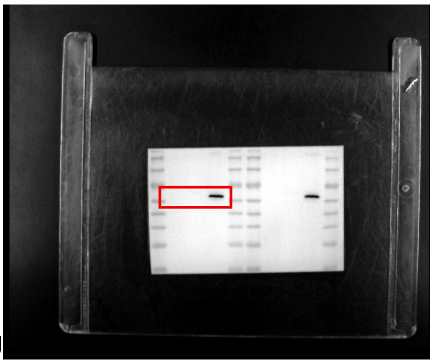

Blot: Flag

Input

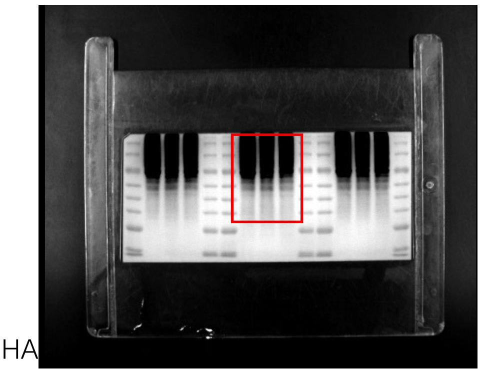

HA

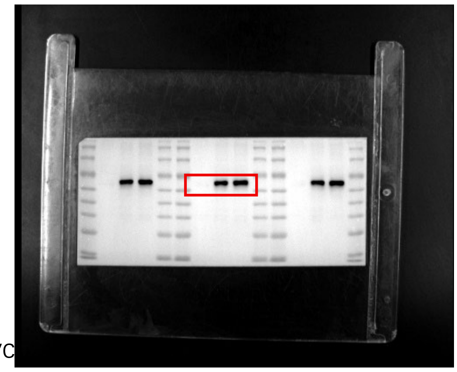

Myc

Flag 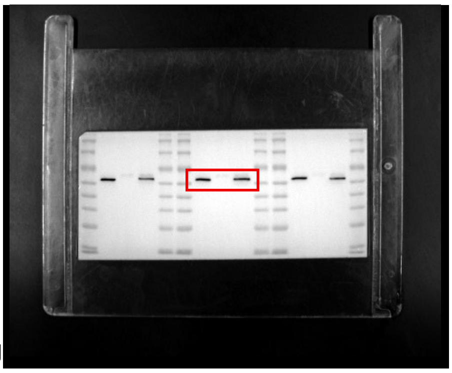 GAPDH 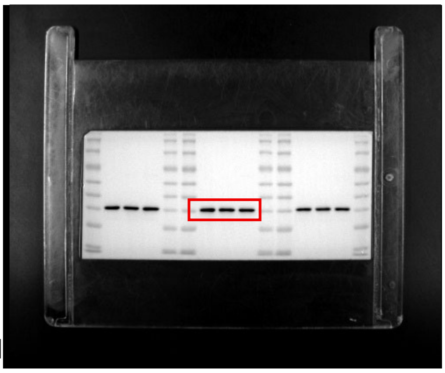

Fig4. B

IP

Blot: HA 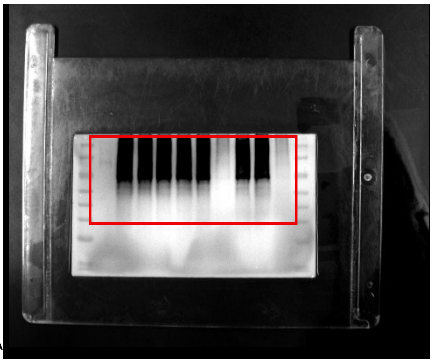 Blot: Myc 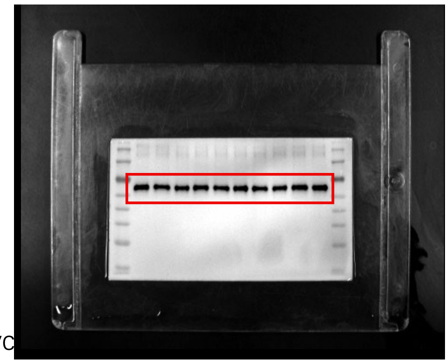

Blot: Flag 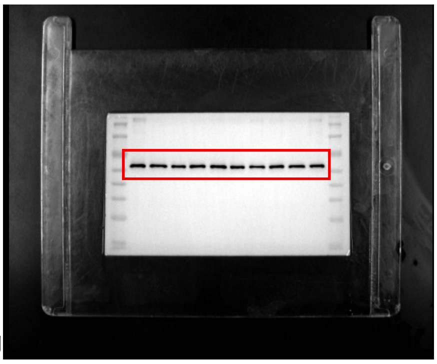

Input

HA 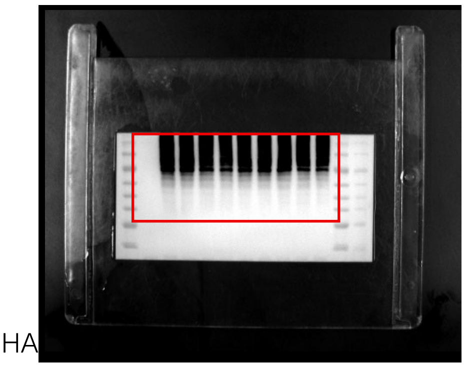 Myc 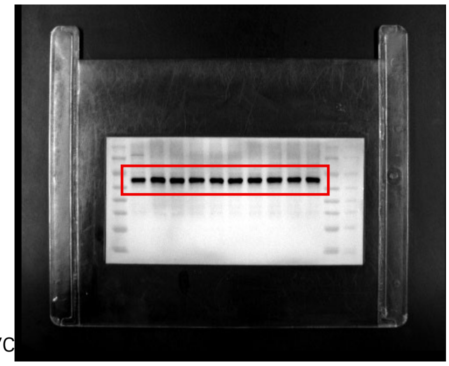

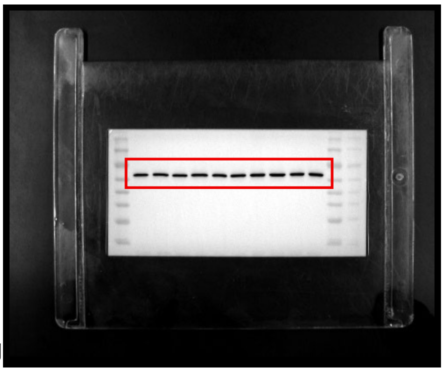

Flag

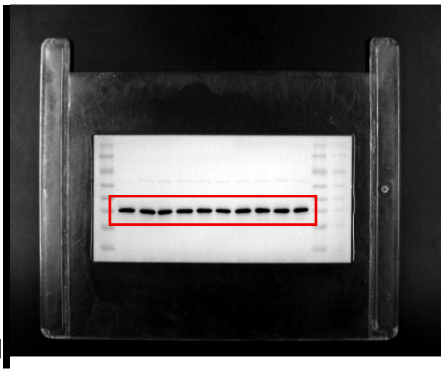

GAPDH

Fig4. C

IP

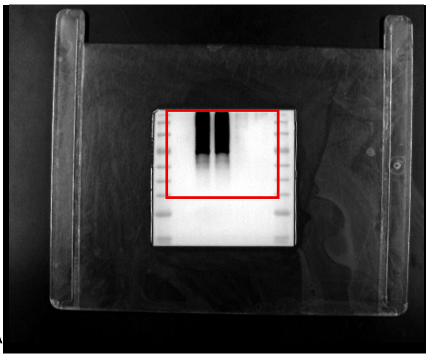

Blot: HA

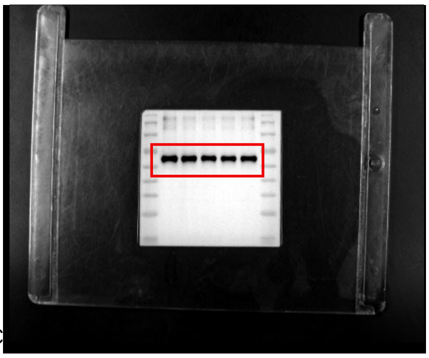

Blot: Myc

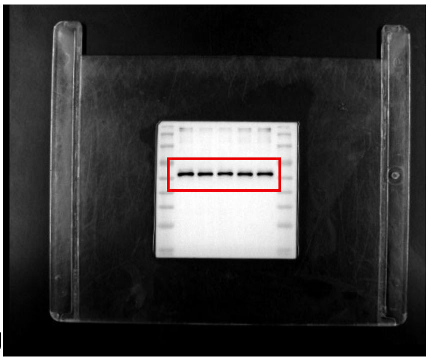

Blot: Flag

Input

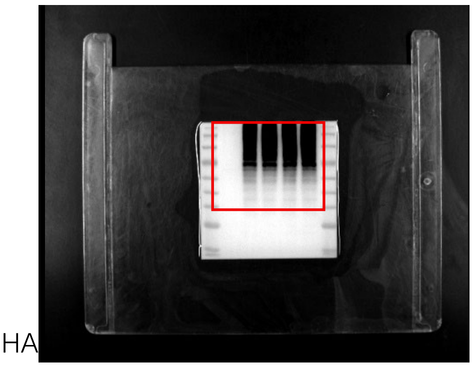

HA

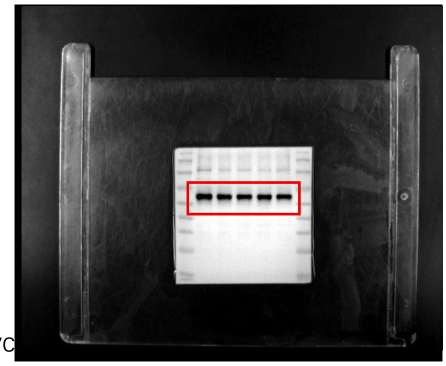

Myc

Flag

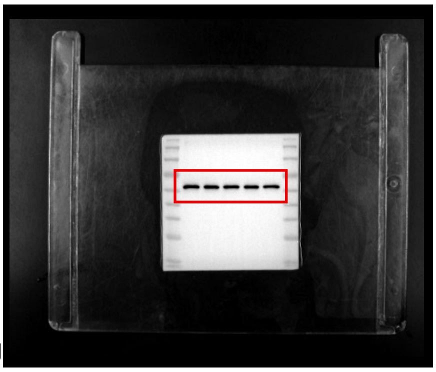

GAPDH

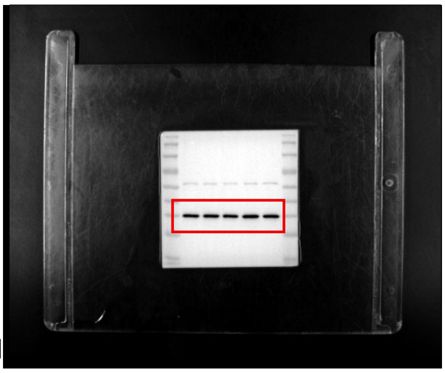

Fig4. E

IP

Blot: Flag

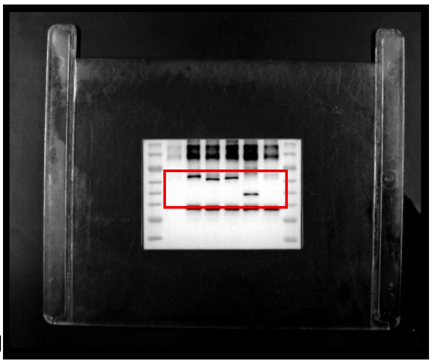

Blot: HA

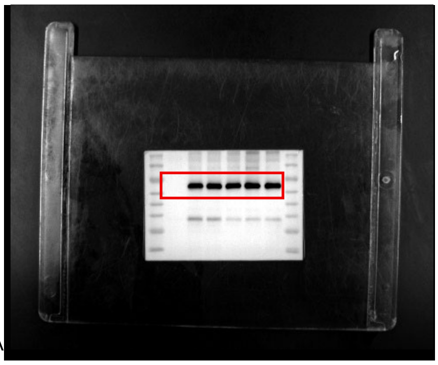

Input

Flag

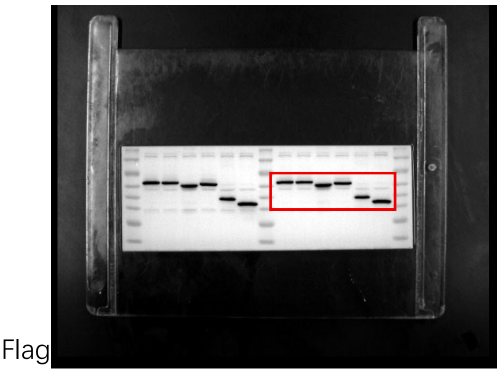

HA

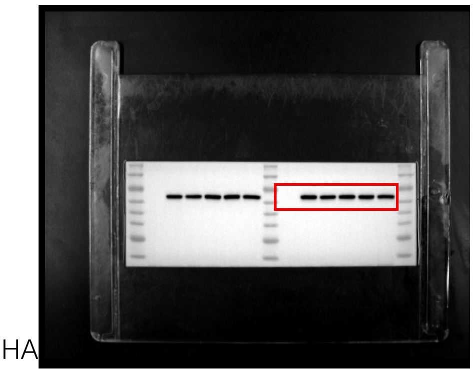

GAPDH

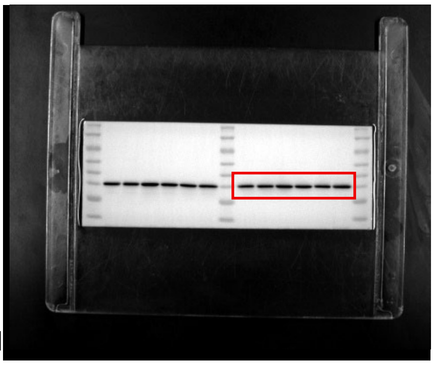

Fig4. H

IP

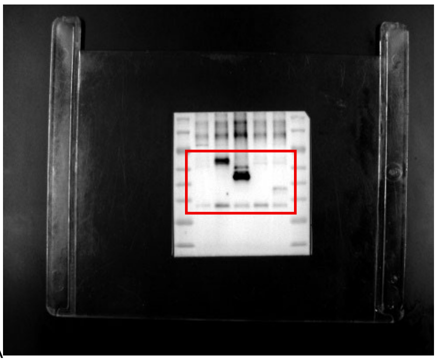

Blot: HA

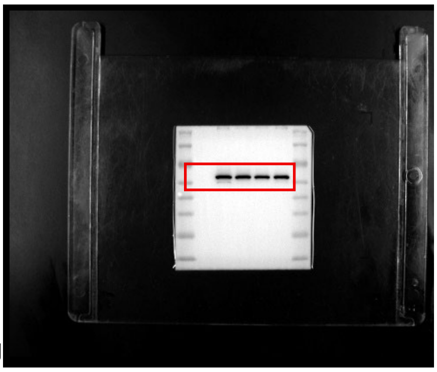

Blot: Flag

Input

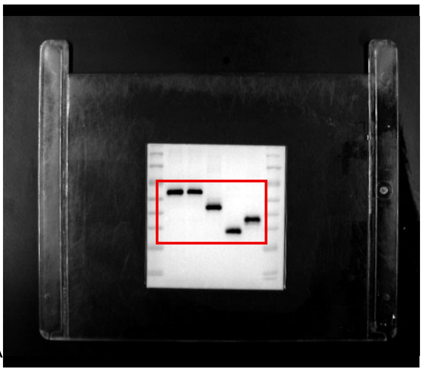

HA

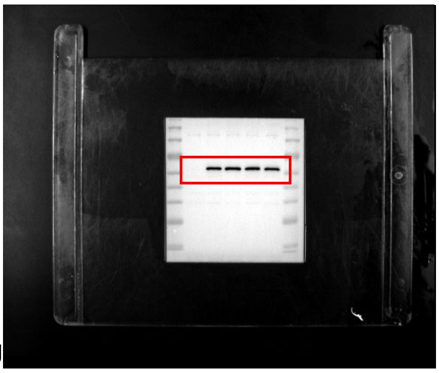

Flag

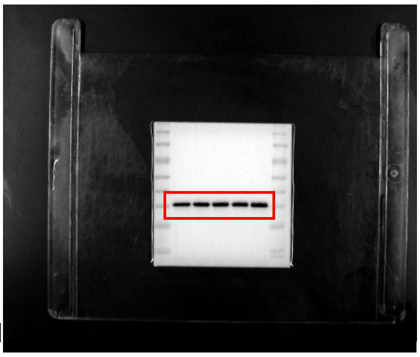

GAPDH

Fig4. J

IP

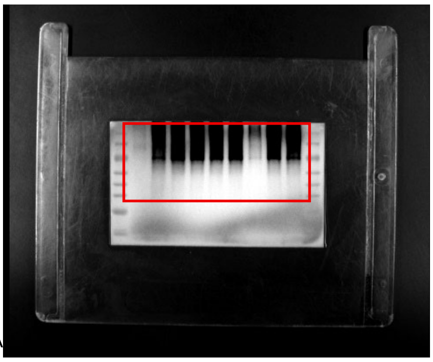

Blot: HA

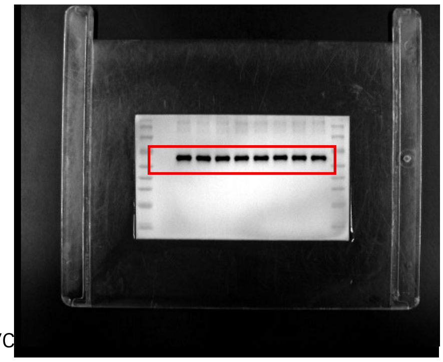

Blot: Myc

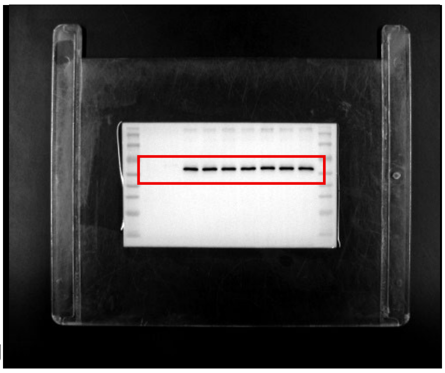
Blot: Flag

Input

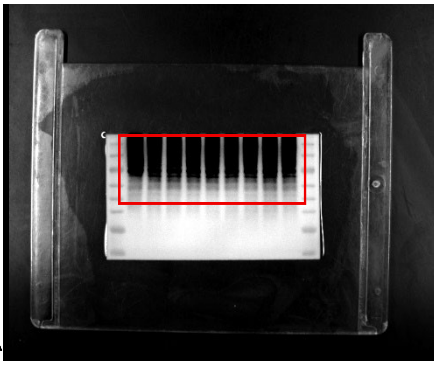
HA

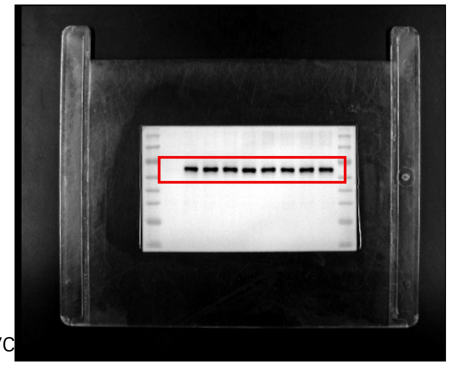
Myc

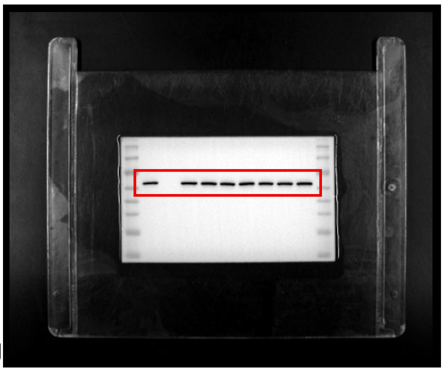
Flag

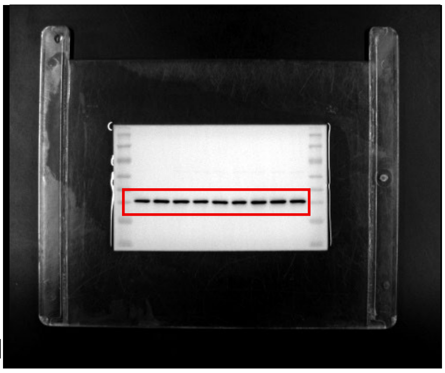
GAPDH

Fig 1. D

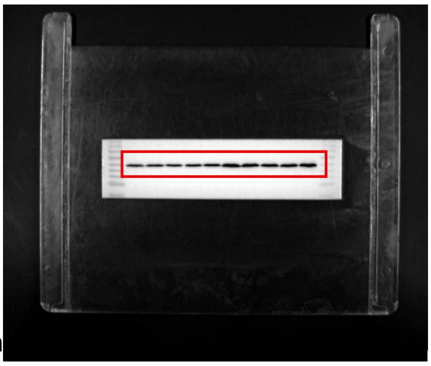
RGMa

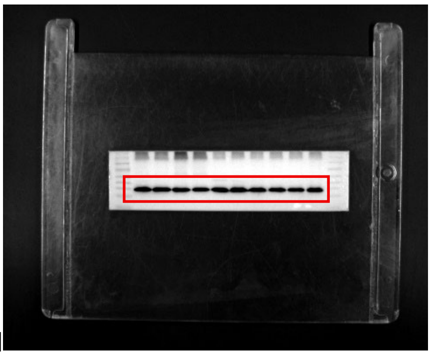
GAPDH

Fig 5. C

RGMa 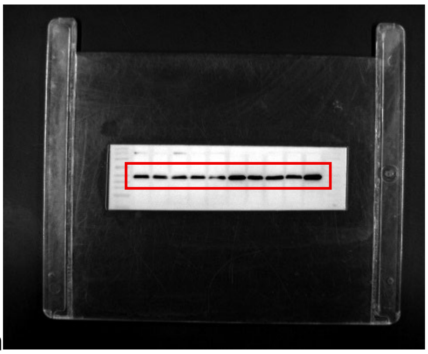

GAPDH 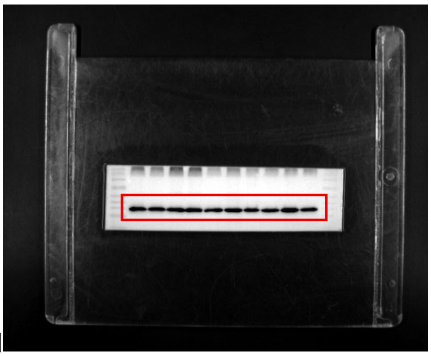

Fig 5. D

RGMa 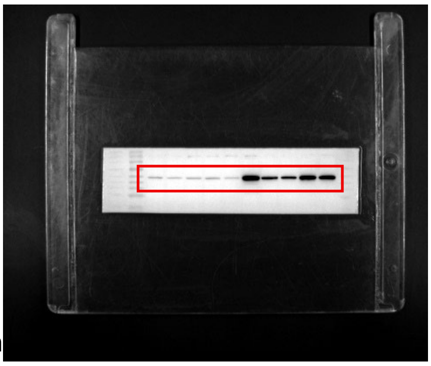

GAPDH 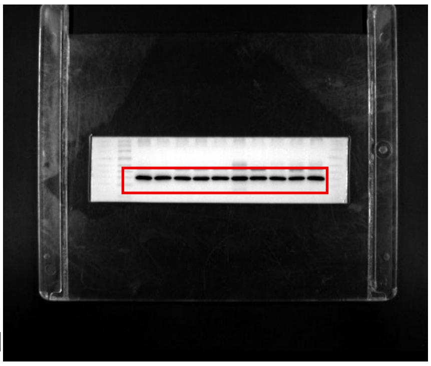

Fig 6. A

RGMa 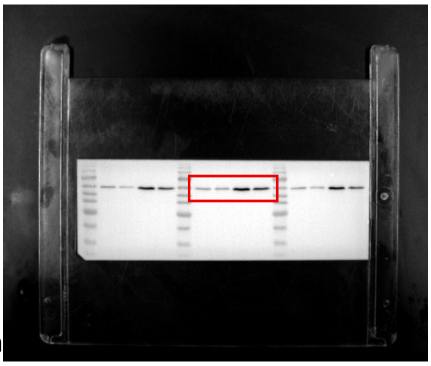

TRIM21 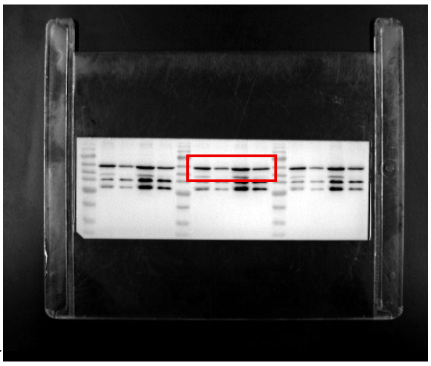

GAPDH 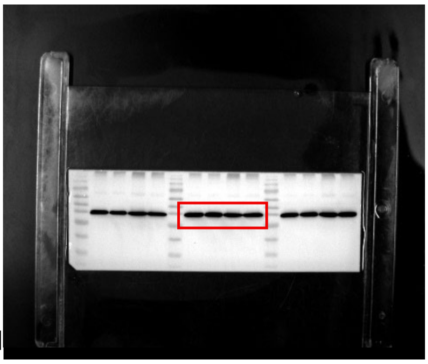

Fig. 7 E

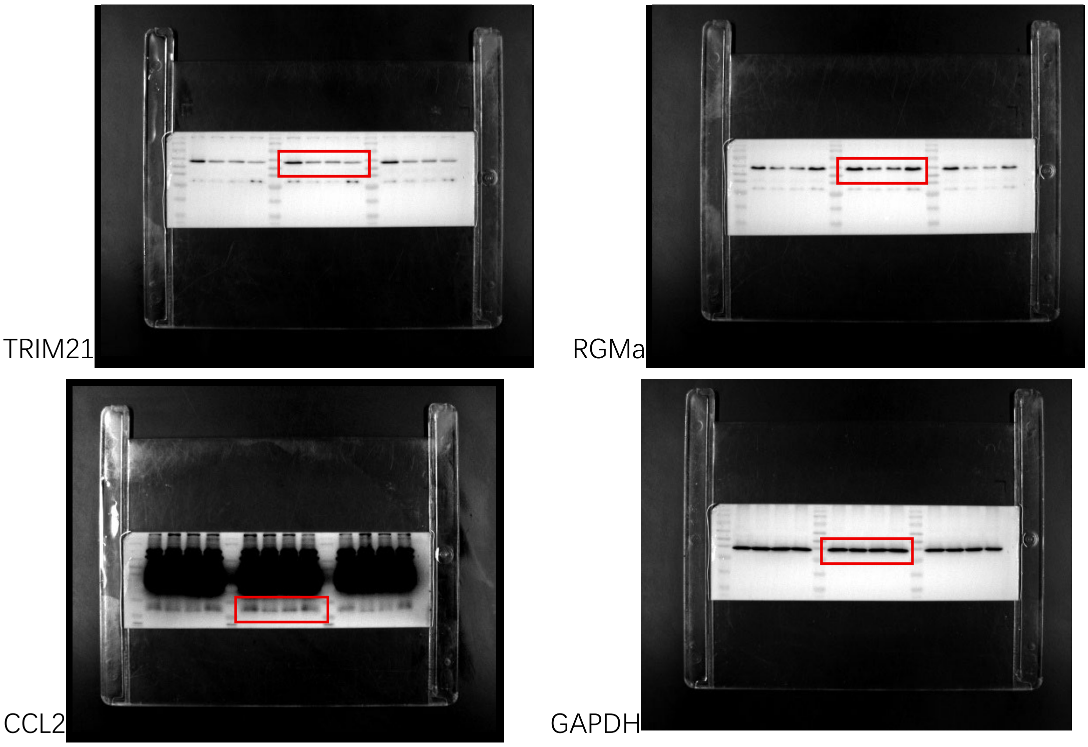

Fig. 7 F

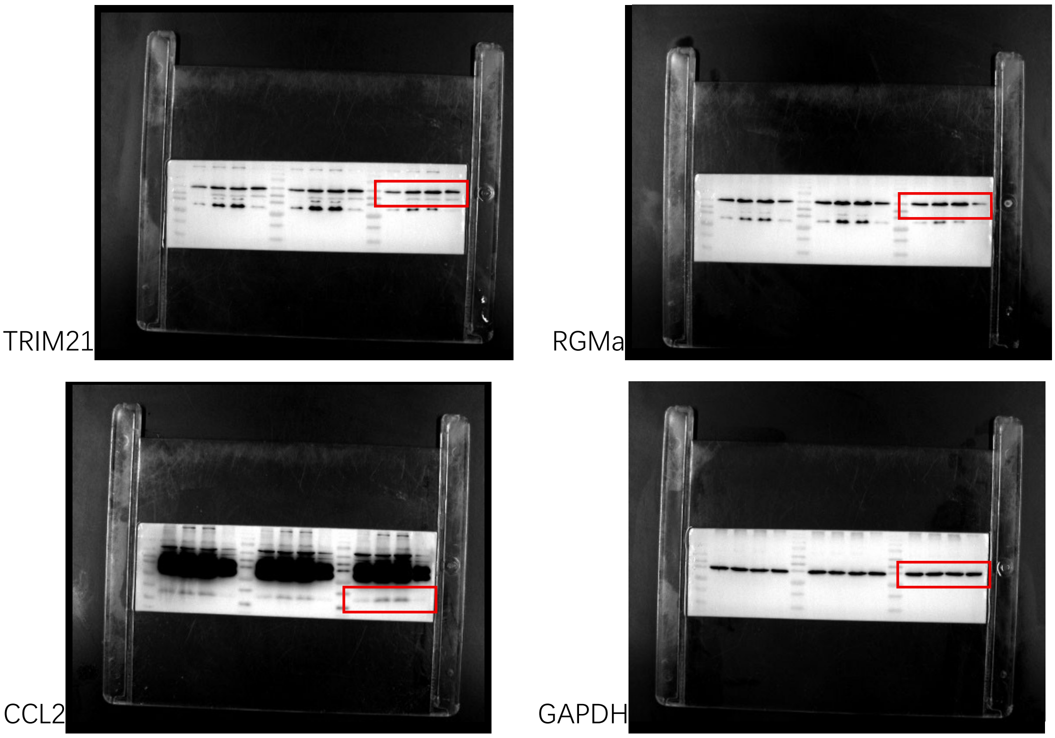

Fig. 8 G

TRIM21
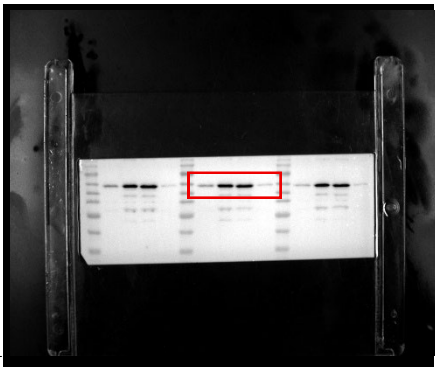
RGMa
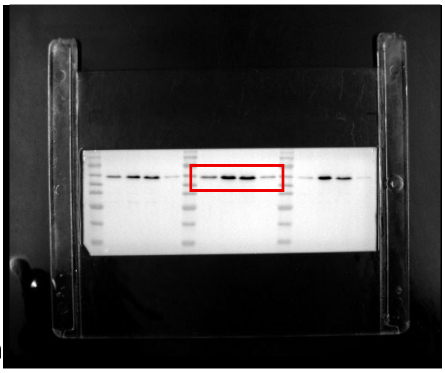
CCL2
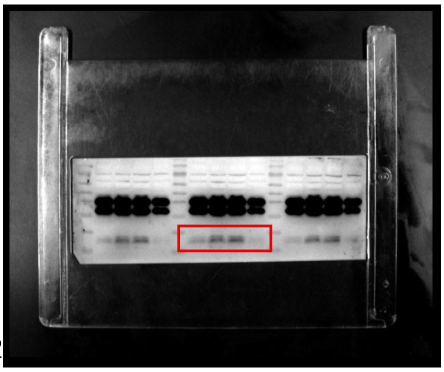
GAPDH
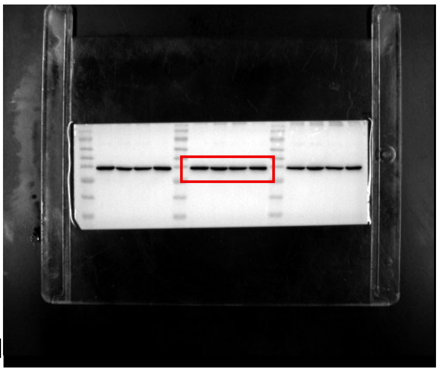

Fig. 8 J

TRIM21
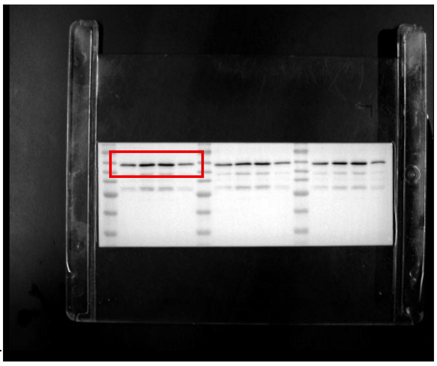
RGMa
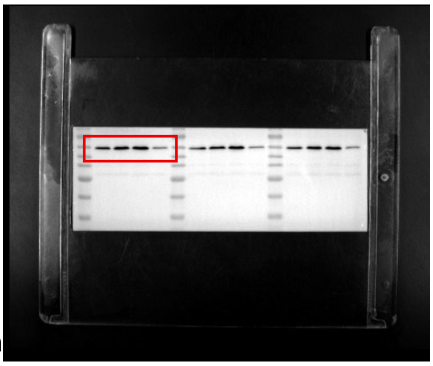
CCL2
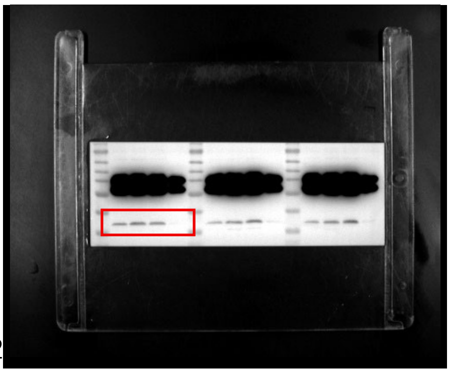
GAPDH
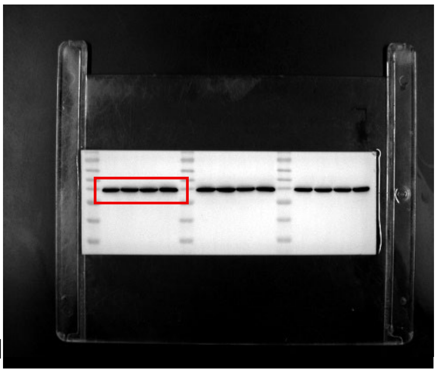

Figure S5

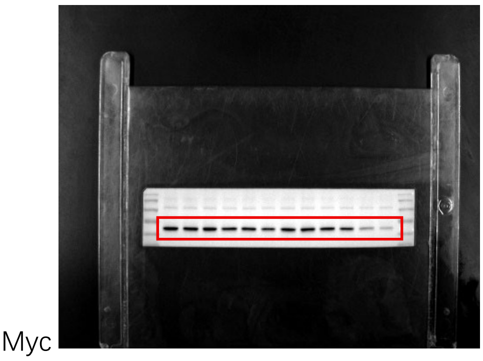

Myc

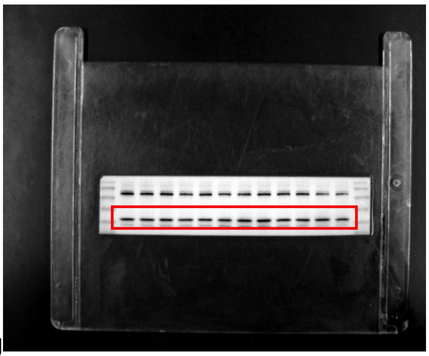

Flag

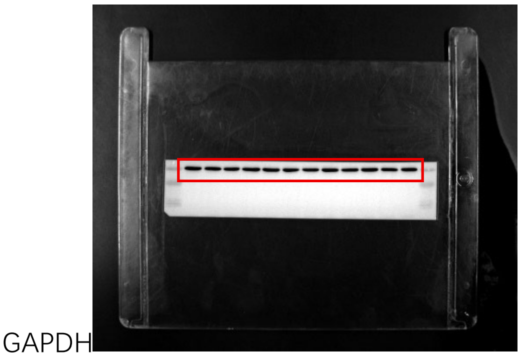

GAPDH

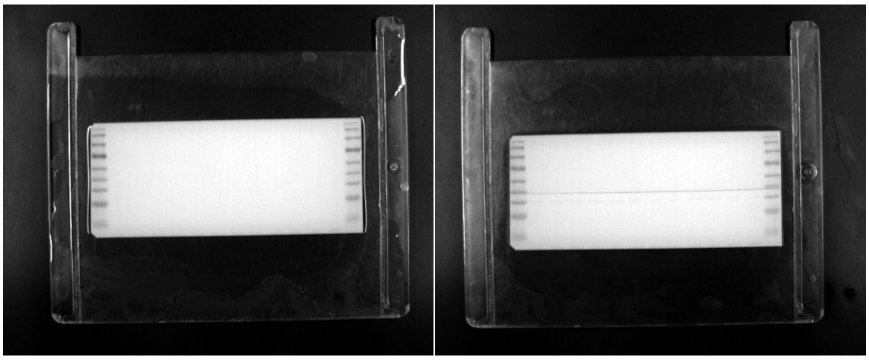

Full membrane

Figure S7 A

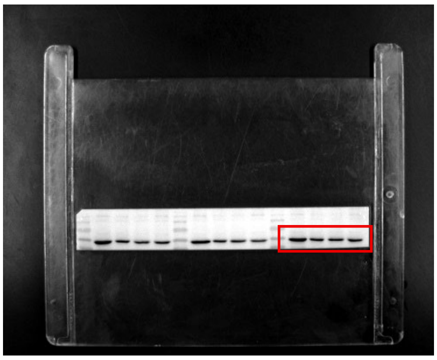

TRIM21

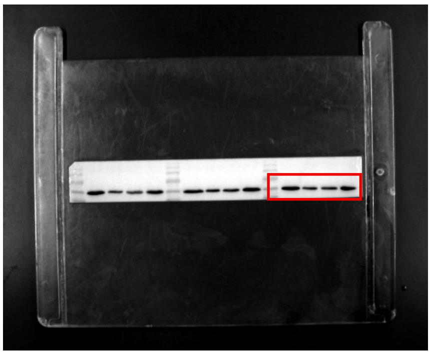

RGMa

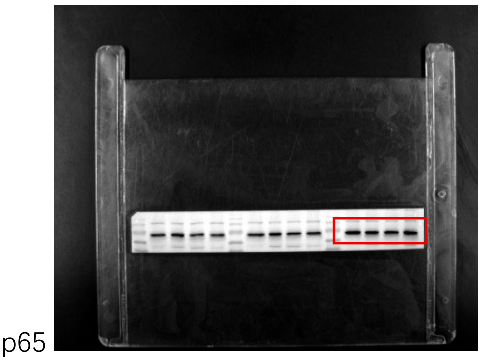

p65

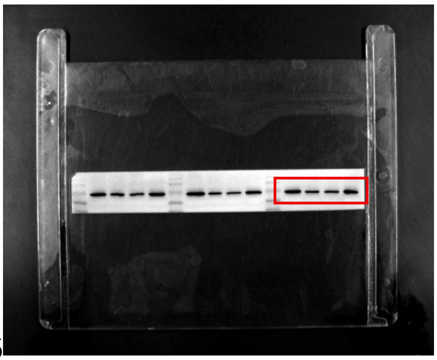

p-p65

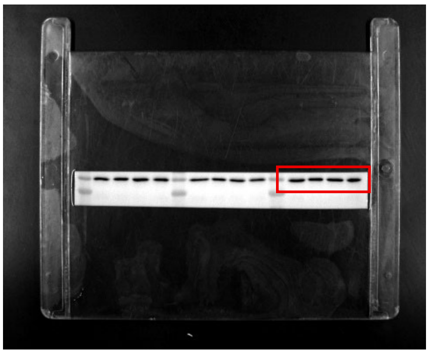

GAPDH

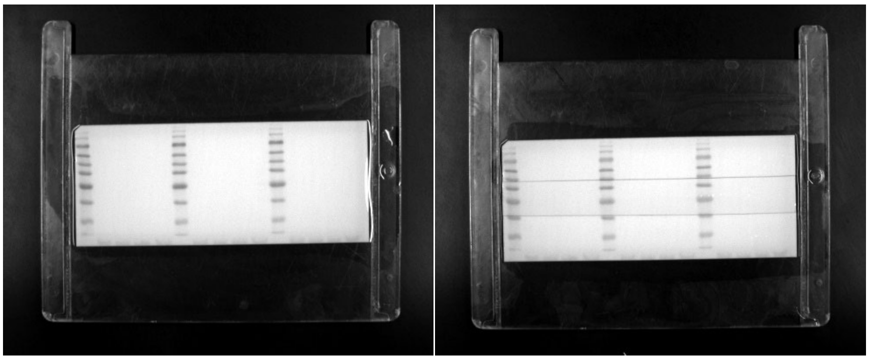

Full membrane

Figure S7 B

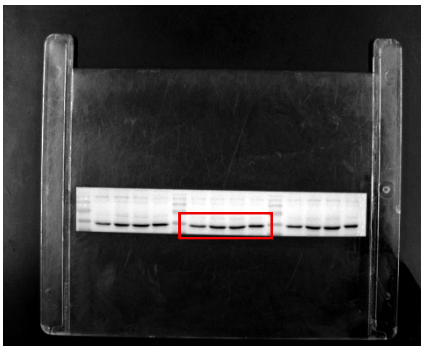

TRIM21

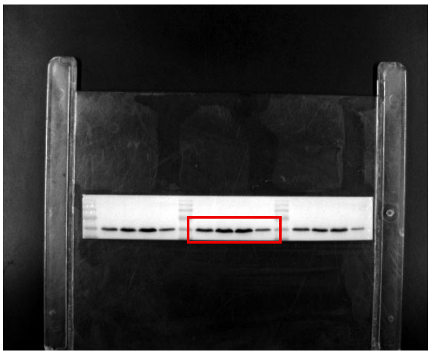

RGMa

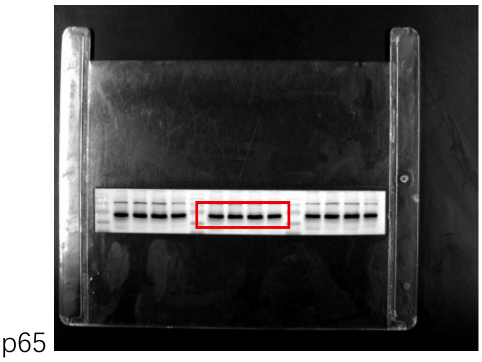

p65

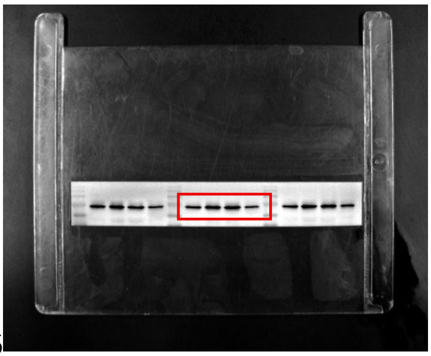

p-p65

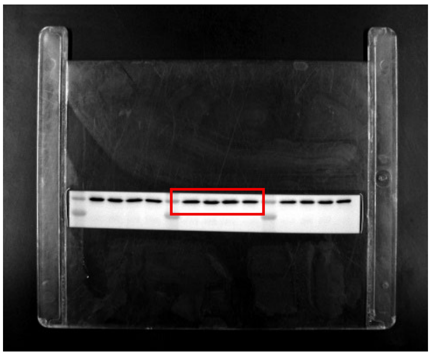

GAPDH

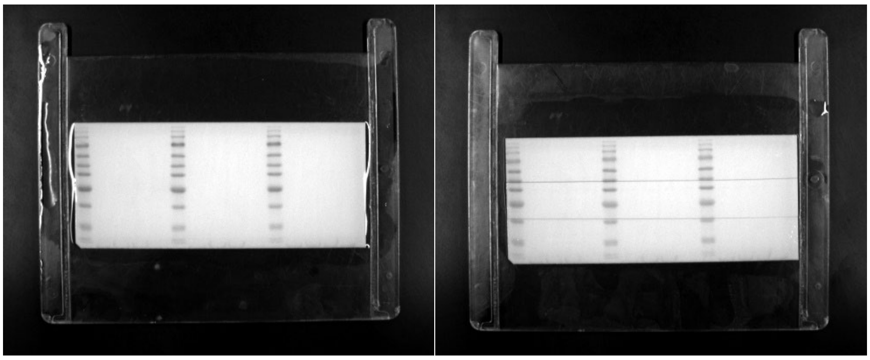

Full membrane

Figure S8

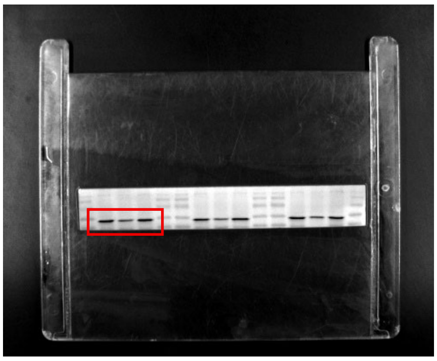

TRIM21

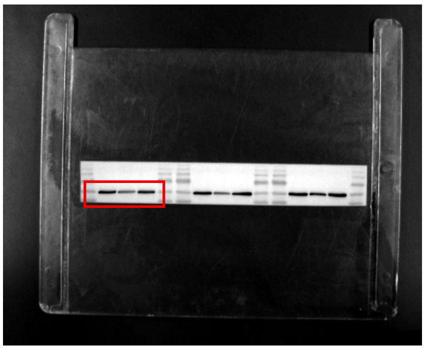

RGMa

CCL2

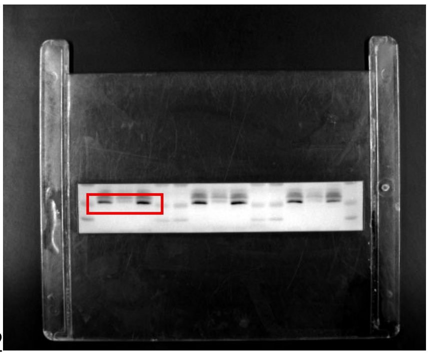

GAPDH

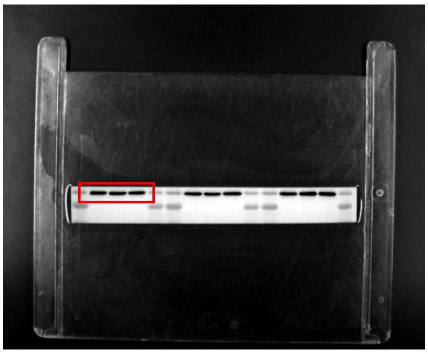

Full membrane

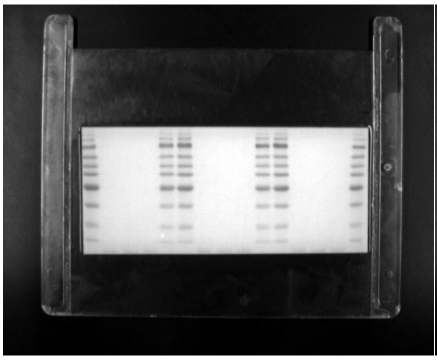

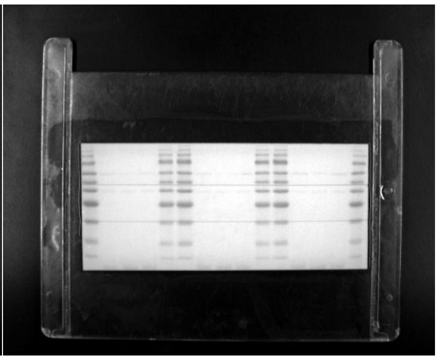

Supplement: Supplementary file 2 — Supplementary Material 2. [file 12974_2026_3769_MOESM2_ESM.pdf]
